# Comprehensive understanding of magnetic hyperthermia for improving antitumor therapeutic efficacy

**DOI:** 10.7150/thno.40805

**Published:** 2020-02-19

**Authors:** Xiaoli Liu, Yifan Zhang, Yanyun Wang, Wenjing Zhu, Galong Li, Xiaowei Ma, Yihan Zhang, Shizhu Chen, Shivani Tiwari, Kejian Shi, Shouwen Zhang, Hai Ming Fan, Yong Xiang Zhao, Xing-Jie Liang

**Affiliations:** 1 Key Laboratory of Resource Biology and Biotechnology in Western China, Ministry of Education; School of Medicine, Northwest University, Xi'an 710069, China; 2 CAS Key Laboratory for Biomedical Effects of Nanomaterials and Nanosafety, CAS Center for Excellence in Nanoscience, National Center for Nanoscience and Technology of China, No. 11, First North Road, Zhongguancun, Beijing 100190, China; University of Chinese Academy of Sciences, Beijing 100049, China; 3 Key Laboratory of Synthetic and Natural Functional Molecule Chemistry of the Ministry of Education, College of Chemistry and Materials Science, Northwest University, Xi'an 710127, China; 4 Beijing General Pharmaceutical Corporation, Beijing 100101, China; 5 The National Institutes of Pharmaceutical R&D Co., Ltd., China Resources Pharmaceutical Group Limited, Beijing 102206, China; 6 Beijing Institute of Traumatology and Orthopaedics, Beijing 100035, China; 7 Neurophysiology Department, Beijing ChaoYang Emergency Medical Center, Beijing 100122, China; 8 National Center for International Research of Biotargeting Theranostics, Guangxi Key Laboratory of Biotargeting Theranostics, Collaborative Innovation Center for Targeting Tumour Theranostics and Therapy, Guangxi Medical University, Nanning 530021, Guangxi Zhuang Autonomous Region, China

**Keywords:** magnetic nanoparticles, magnetic hyperthermia, macroscopic heating, local induction heat, synergistic strategy

## Abstract

Magnetic hyperthermia (MH) has been introduced clinically as an alternative approach for the focal treatment of tumors. MH utilizes the heat generated by the magnetic nanoparticles (MNPs) when subjected to an alternating magnetic field (AMF). It has become an important topic in the nanomedical field due to their multitudes of advantages towards effective antitumor therapy such as high biosafety, deep tissue penetration, and targeted selective tumor killing. However, in order for MH to progress and to realize its paramount potential as an alternative choice for cancer treatment, tremendous challenges have to be overcome. Thus, the efficiency of MH therapy needs enhancement. In its recent 60-year of history, the field of MH has focused primarily on heating using MNPs for therapeutic applications. Increasing the thermal conversion efficiency of MNPs is the fundamental strategy for improving therapeutic efficacy. Recently, emerging experimental evidence indicates that MNPs-MH produces nano-scale heat effects without macroscopic temperature rise. A deep understanding of the effect of this localized induction heat for the destruction of subcellular/cellular structures further supports the efficacy of MH in improving therapeutic therapy. In this review, the currently available strategies for improving the antitumor therapeutic efficacy of MNPs-MH will be discussed. Firstly, the recent advancements in engineering MNP size, composition, shape, and surface to significantly improve their energy dissipation rates will be explored. Secondly, the latest studies depicting the effect of local induction heat for selectively disrupting cells/intracellular structures will be examined. Thirdly, strategies to enhance the therapeutics by combining MH therapy with chemotherapy, radiotherapy, immunotherapy, photothermal/photodynamic therapy (PDT), and gene therapy will be reviewed. Lastly, the prospect and significant challenges in MH-based antitumor therapy will be discussed. This review is to provide a comprehensive understanding of MH for improving antitumor therapeutic efficacy, which would be of utmost benefit towards guiding the users and for the future development of MNPs-MH towards successful application in medicine.

## Introduction

Magnetic hyperthermia (MH) can be dated back to 1957, where Gilchrist *et al.* selectively heated the tumors by exploiting magnetic particles in the presence of an alternating magnetic field (AMF) 1. With the rise in nanotechnology, the introduction of magnetic nanoparticles (MNPs) has further evolved this approach into a well-researched field 2. The most significant advantage of MNPs-mediated MH (MNPs-MH) therapeutic modality is in the deep tissue penetration and magselectively killing of cancer cells without harming the surrounding healthy tissues 3-6. MNPs-MH helps in realizing intracellular hyperthermia 7, as it directly delivers therapeutic heating to the cancer cells, and this intracellular hyperthermia can be further improved by conjugating the cell-targeting ligands with MNPs. The local and homogeneous heat leads to the greater selectivity and effectiveness of the treatment. Due to all these therapeutic benefits, MNPs-MH based tumor treatments have recently been translated from the lab to clinical trials, and they have been used for the treatment of glioblastoma and prostate cancer 8. Many clinical trials were further performed by MagForce (Berlin, Germany), to investigate MNPs-MH therapy for pancreatic cancer. Although MNPs-MH therapy has been conducted in clinical trials, further research and development work are required to realise the full potential of this cancer nanotechnology. In particular, significant hurdles, such as the effectiveness and efficiency of MNPs-MH modality in cancer therapy have to be researched on, have to be overcome which posed challenges to this treatment modality.

Generally, MNPs-MH therapy primarily involves the raising of tumor local temperature within the range of 43-46 ºC, resulting in the changed physiology of the cancer cell, which eventually leads to their apoptosis/necrosis 3, 6, 9. To make MNPs-MH clinically useable and cancer eradication feasible, it is essential that sufficient heat must be delivered within the whole tumor mass while leaving the surrounding normal tissues unaffected. As such, a strict limitation in *H* and *f* of AMF where *H*×*f* < 5×10^9^ for biomedical reasons is imposed 10. With this limitation in AMF, the therapeutic efficacy of MNPs-MH hence depends on the thermal conversion efficiency of MNPs. Among several MNPs, superparamagnetic iron oxide nanoparticles (SPIONs) are extensively investigated as MNPs-MH agents due to their high biocompatibility/biodegradability. However, their insufficient thermal conversion efficiency attributed to the degraded magnetic susceptibility has been portrayed as a critical problem in practical applications. Thus, to address such issue, significant research efforts have been devoted into the improvement of magnetic susceptibility so as to achieve drastically enhanced induction heating properties, with introduced strategies such as by modulating the particle size 11-19, controlling composition 20-27, manipulating the shape 17, 20, 28-32, and modifying surface 33. Despite the availability of these strategies, there is an eventual limit to the thermal conversion efficiency of the MNPs due to their intrinsic non-resonance absorption nature under an AMF. Apart from this well-established macroscopic heating effect, recent works have suggested that a single MNP has the capability to raise the local temperature of the molecules either attached to or in the vicinity of MNP. This instantaneous nanoscale heat can regulate the physiological and biochemical properties of molecules and cause functional changes in organisms 34, 35. The biological effect of localized induction heat might inject new vitality into the enhancement of antitumor therapeutic efficacy of the MNPs-MH 7, 36-38. For example, under AMF, lysosome-accumulated MNPs would induce lysosomal cell death by increasing the local temperature and enhancing reactive oxygen species (ROS) production within the lysosomes 39, 40. The biological effects of local induction heat in cellular/subcellular death and its controllable regulation mechanisms still require a significant amount of research and study. However, due to the shallow understanding of MNPs-MH, progress in this area is slow with limited new advancements that lead to finite development in this field. Additionally, MNPs-MH was often exploited as a combinatorial approach to enhance the sensitivity of traditional cancer treatment methods such as chemotherapy, radiotherapy, immunotherapy, photothermal/photodynamic therapy (PDT), and gene therapy. Such a combinatorial approach has been rapidly realized as a strategy to improve the tumor treatment efficacy, which poses a potential alternative therapeutic modality for cancer treatment.

**Figure 1:**
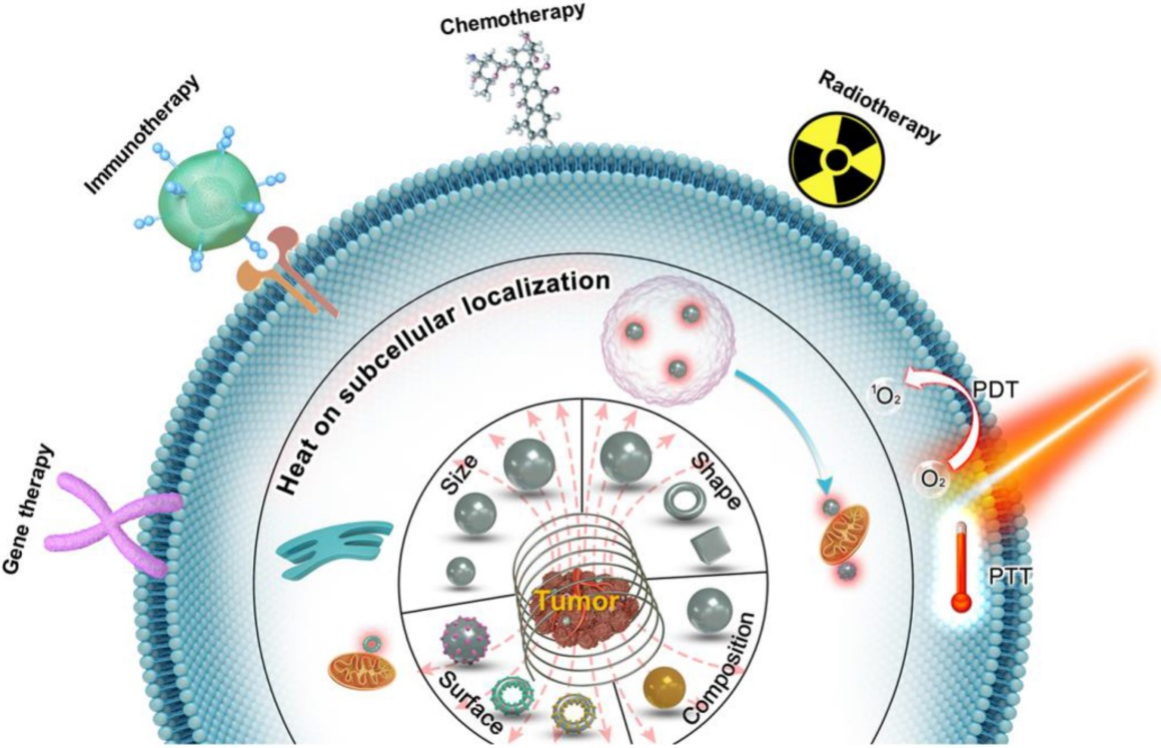
Schematic showing the strategies for improving antitumor therapeutic efficacy of MNPs-MH therapy, (1) by optimization of size, composition, morphology, and surface of MNPs to render sufficient and effective heating properties, (2) deep understanding of the effects of localized induction heat for the disruption of cellular/subcellular structures, which enhances the efficacy of MNPs-MH in improving antitumor therapeutic therapy, and (3) by synergistically combining MNPs-MH with other therapeutic strategies, such as chemotherapy, radiotherapy, immunotherapy, photothermal/PDT, and gene therapy, lallows further enhancement of the antitumor effectiveness.

The adoption of various strategies for improving the antitumor therapeutic efficacy of MNPs-MH therapy will be focused in this review (Figure 1). Firstly, the effects of the MNPs design on their thermal conversion efficiencies will be discussed. Next, the recent advances in MNPs-MH will be presented in 2 parts; (1) the induction of perceptible temperature rise by magnetic hysteresis/relaxation of MNPs, and (2) the effects of local temperature elevation in cancer cell death. The methods for measuring the temperature at the MNPS surface with subnanometer resolution will also be described in this review. Furthermore, a synergistic strategy that involves the combination of MNPs-MH with other modalities such as chemotherapy, radiotherapy, immunotherapy, photothermal/PDT, and gene therapy *et al*. will be elucidated to understand its mechanism towards the enhanced antitumor efficacy. Finally, the perspectives and challenges in MNPs-MH will be explored. The aim of the review is to present the available strategies to enhance the effectiveness of the antitumor treatment and to provide a systematic and comprehensive review of MNPs-MH.

## Macroscopic heating effects of MNPs

As one of the most critical components in MNPs- MH, it is highly necessary for MNPs to be safe and highly efficient. MNPs are magnetic nanomediators that mediate the conversion of electromagnetic waves to thermal energy. As such, to improve the therapeutic efficacy of MNPs-MH, the most fundamental strategy is to increase the thermal conversion efficiency of MNPs. The inductive heating effect of MNPs, when subjected to an AMF, can be affected by numerous factors, such as hysteresis effect, relaxation effect, eddy current, domain wall, natural resonance, and so on 41. When the strength and frequency of AMF are insufficient to cause significant eddy current, macroscopic thermal efficiency of MNPs should be closely related to their intrinsic physicochemical properties.

### Magnetic loss of MNPs

**Figure 2:**
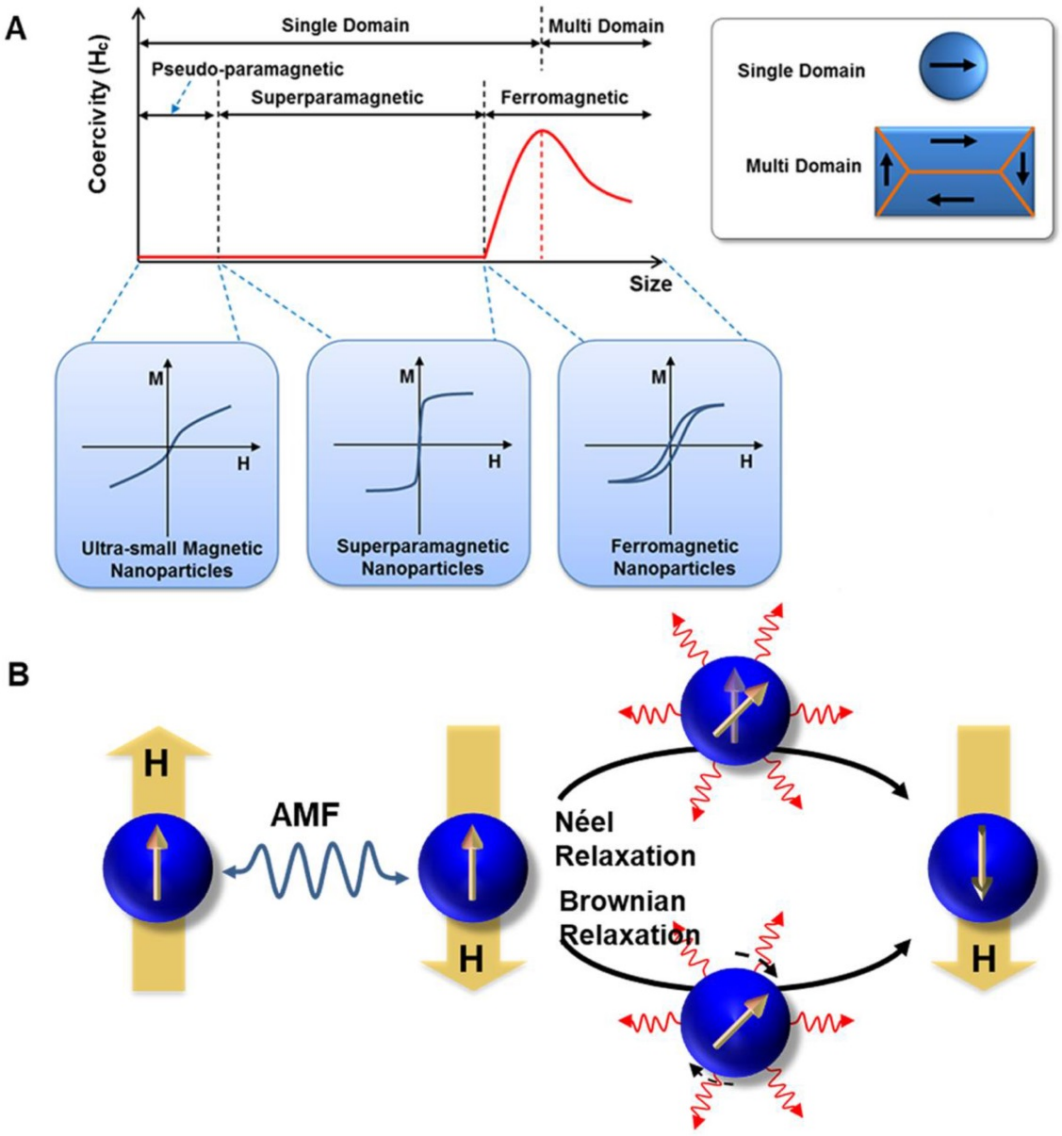
** A.** Representation of typical hysteresis loops of a ferromagnetic/ferrimagnetic nanomaterial and a superparamagnetic nanomaterial and, and the dependence of coercivity on particle size. **B.** Néel and Brownian relaxation mechanisms.

Magnetic loss of MNPs is the amount of AMF energy converted into heat during magnetization reversal. Magnetic losses largely depend on the magnetic behavior of MNPs that is highly size-dependent (Figure 2a) 5, 19, 42-44. In bulk material, they usually exist in a multi-domain state. However, as the size of the domain is reduced, the maintenance of the domain wall becomes too energy-intensive and therefore leading to the formation of a single domain MNPs. Below a certain size, the thermal energy overcomes the anisotropy energy, preventing stable magnetization and resulting in superparamagnetic behavior. For typical magnetic iron oxide nanoparticles (MIONs), the critical size limit to achieve SPIONs is around 25 nm at room temperature 6. When ferromagnetic/ ferrimagnetic MNPs are placed in an AMF, there is a magnetic hysteresis loss.

The amount of heat produced by the MNPs approximately equals to the area of the hysteresis loop, during one cycle of the magnetic field 45. The heat dissipation of both single-domain and multi-domain NPs always derived from hysteresis losses, which depends on how fast the magnetization follows the AMF changes. For superparamagnetic nanoparticles with a particle size smaller than the single-domain range, the heat dissipation mechanism mainly comes from the energy loss caused by overcoming the rotational energy barrier under AMF 19, 43, 45. Under AMF, the rotations need to overcome the friction between magnetization easy axis and atomic lattices for Néel relaxation or between MNPs and their surroundings for Brownian relaxation, leading to the loss of electromagnetic energy and the production of thermal energy (Figure 2b). Néel and Brownian mechanisms describe the relaxation (magnetization rotations) of single-domain particles (superparamagnetic as well as ferromagnetic NPs). The only difference between single-domain ferromagnetic and superparamagnetic NPs is that the latter randomly change their magnetization direction due to thermal fluctuations in the time scale of quasi-static magnetic measurements, displaying zero remanence and coercivity.

### Experimental techniques for magnetic heating efficiency evaluation

#### Calorimetry

The magnetic heating efficiency of MNPs is generally evaluated by specific heat absorption rate (SAR) or their equivalent parameters-specific loss power (SLP), or intrinsic loss power (ILP) 46, 47.


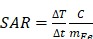



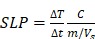
 Equation (1)

where *c* is the volumetric specific heat capacity of the solution, m_Fe_ is the mass concentration of magnetic element in the solution, m is the mass of magnetic element in the solution,* Vs* is the sample volume. Removing *H* and *f* factors, it can be as ILP:


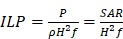
 Equation (2)

where ρ is the density of nanomaterial. ILP is independent of the magnetic field parameters and can be used to compare the heating conversion efficiency of MNPs measured under different magnetic fields.

Calorimetry is a straightforward technique in measuring the amount of heat so as to determine SAR. Based on the measurement of temperature evolution of the target sample, heat quantification can be achieved. Since the temperature progress of the sample is the result of the balance between heat generated by the sample and heat exchange with the environment, it is necessary to have a set-up that measures temperature and a thermal model that describe such a physical system. The thermal properties and geometric characteristics of the sample and its environment can also influence the measurement. Current SAR installations reported in the literature 33, 42, 48 consist of 4 main components; (1) an AMF generator (2) a sample chamber that is defined by isolating material, (3) temperature sensors, and (4) a data acquisition system. Since heat losses (based on conduction, radiation, and convection) are not minimized, such setups do not provide adiabatic conditions. To estimate the SAR, the temperature-versus-time exponential curve was used, according to Equation (1), where 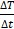 is the initial slope. If there are significant initial thermal losses or inhomogeneous temperature across the sample, such a procedure can result in unknown errors in the determination of 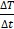 . Thus, in order to minimize the errors, 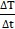 is calculated as the maximum slope of the time-dependent temperature curve 45, 49, 50.

#### Alternating current (AC) magnetometry

The SAR obtained from different types of magnetic measurements can be determined from the relationship between the magnetic response of MNPs and their generated heat. The changes in magnetic flux density resulted in the sample over time can be measured by quantifying the current induced in a gradiometric inductive coil.

A so-called hysteresis loop is observed as a ferro/ferrimagnetic material is exposed to an AMF. This hysteresis loop refers to the non-linearity and delay of its magnetization with respect to the applied field, *H*, originating from the M versus *H* trend. Heat dissipated per AMF cycle can be determined from the area entrapped within the M(*H*) cycle, and SAR can be determined from the quantification of this area according to the following equation 19:


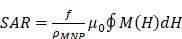
 Equation (3)

where 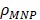
is the density of the magnetic material, µ_0_=4π×10^-7^ T m/A is the permeability of free space, and *M* and *H* are expressed in SI units (A/m). Based on Equation (3), SAR can be determined using magnetic methods by integrating the experimental *M(H)* curve and taking into account the AMF frequency to obtain the area of the hysteresis loop between ±*H_0_*.

In general, even though magnetic methods are accurate in determining the SAR, they do not operate under the MH conditions. As most of the SAR determination experiments are performed with pairs (*f*, *H_0_*), they are not within the biological range. Meanwhile, in the case of magnetic methods, due to the lack of experimental set-ups that can work under AMF parameters suitable for clinical MNPs-MH, SAR determination is mostly inaccurate 51.

#### Infrared/Fluorescence thermometry

Temperature sensing and imaging by infrared thermometry is a common method for experimental thermometry of samples, cells, or animal surfaces. However, the relative orientation (in terms of angle) between the measured surface and infrared camera can strongly influence the magnitude of the surface temperature measured by infrared thermometry. This, in turn, translates to the possibility of affecting the temperature measurements when there are any changes in the relative position of the small animal during the *in vivo* procedures 51. Thus, infrared thermometry cannot evaluate accurate intratumoral temperature.

Fluorescence thermometry is usually used in measuring cellular temperature because it can operate as “non-contact” devices and provide temperature sensing at the nanoscale. Attaching a fluorophore to the shell of the MNPs via a bond that breaks at a particular temperature 52, and then analyzing the fluorophore content in the supernatant after heating can lead to the determination of the bond-breaking temperature. This is an indirect method of magnetic heating efficiency, which is distant from producing a real-time and continuous temperature reading.

### Strategies for improving SAR

Plenty of research studies have shown that the SAR of different MNPs types may vary by orders of magnitude, which is dependent on its intrinsic magnetic properties and extrinsic magnetic field amplitude 53-55. For biomedical safety reasons, there is a strict limitation for AMF where *H×f* < 5×10^9^ 43. Currently, due to the dependency of SAR on the magnetic properties of the MNPs, significant researches have been focused on improving the SAR by selecting appropriate magnetic material, controlling/regulating particle core size, composition, shape, and surface shell whereby all of these factors can affect the magnetic susceptibility, saturation magnetization (*M_s_*), magnetocrystalline anisotropy (*K*), and relaxation time 5, 56, 57. The magnetic susceptibility/*M_s_* of MNPs decreases rapidly as the size decreases due to the reduced *K* and spin disordering on the surface of MNPs, while larger-sized MNPs usually have higher magnetic susceptibility/*M_s_* than that of smaller-sized MNPs. Thus, this suggests that SAR value increases as size increases, which reach a maximum value at a specific size 19, 42. SAR is proportional to *M_s,_* but it is inversely proportional to the size distribution of MNPs 13, 14. The SAR value can have a maximum point at a certain particle size, and *K* 19. Therefore, MNPs with high *M_s_* and high mono-dispersivity are desirable for effective magnetic loss.

**Figure 3:**
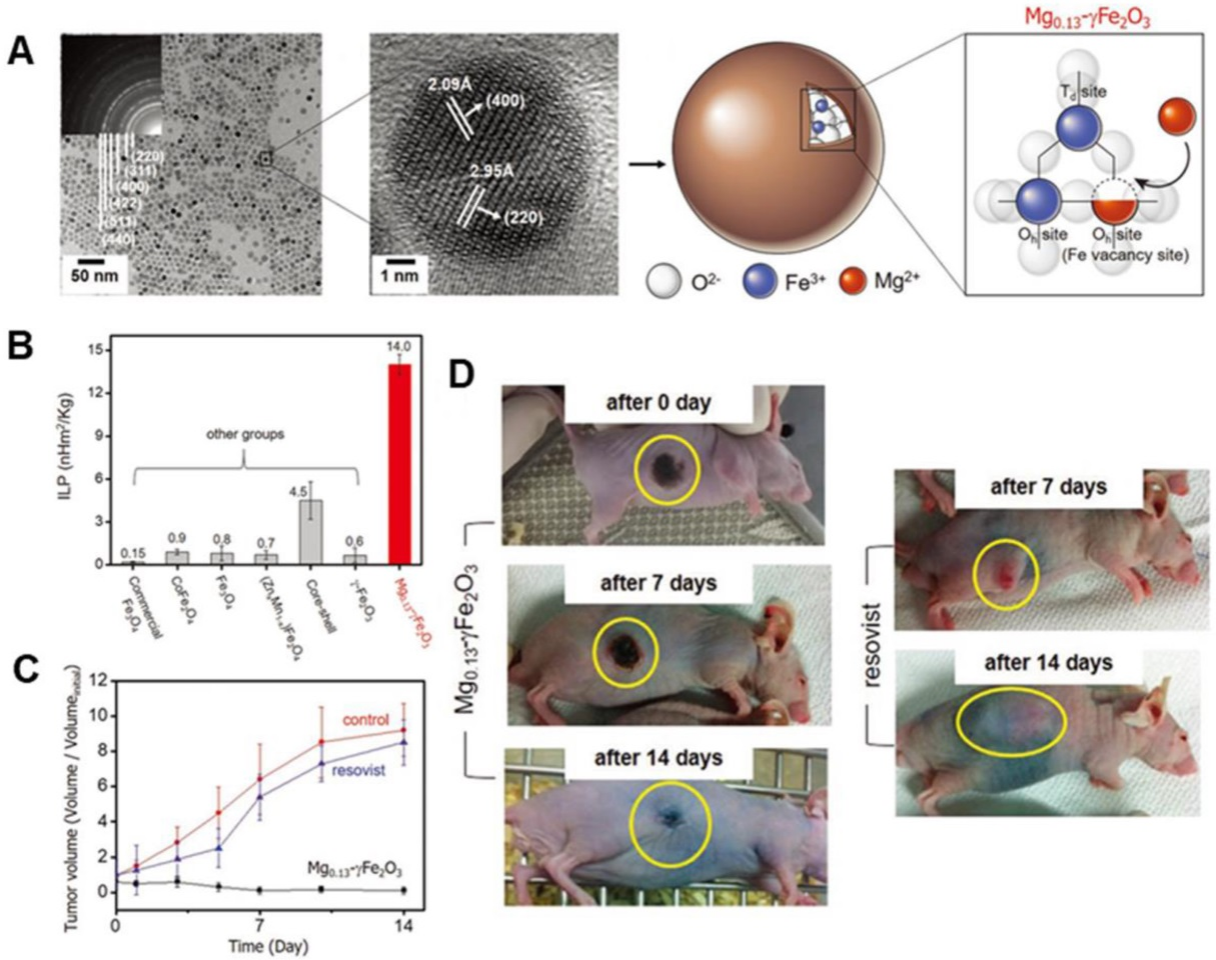
** A.** TEM image of Mg_0.13_-γFe_2_O_3_ NPs (Inset image showing selected area electron diffraction patterns). High-resolution TEM image of Mg_0.13_-γFe_2_O_3_ NPs in the middle and a schematic diagram of spinel structure of Mg_0.13_-γFe_2_O_3_ NPs in the right. **B.** Comparison of ILP value of as- developed Mg_0.13_-γFe_2_O_3_ NPs to other previously reported SPIONs. Two types of commercial Fe_3_O_4_ (Feridex and Combidex) can be used for baseline. **C.** Dependence of tumor volume change (*V/V*_initial_) on time after MH with untreated control, Resovist, and Mg_0.13_-γFe_2_O_3_ nanofluids. **D.** Photographs of xenografted nude mice after MH with Mg_0.13_-γFe_2_O_3_ nanofluids and Resovist. Reproduced with permission from ref 24. Copyright 2017 John Wiley & Sons Ltd.

Besides the size of MNPs, the composition and shape of MNPs are also essential factors in modulating the magnetic property 58-61. Table 1 summarizes the composition and shape effect of MNPs on SAR. Regulating the composition of MNPs is an effective way to modify their magnetism so as to enhance the SAR, *e.g.*, metallic MNPs (*i.e.*, Fe, Co, FeCo) with high *M_s_* could achieve high SAR 62. However, despite their high SAR, Fe or FeCo NPs are usually unstable in ambient conditions. Lacroix *et al.* reported Fe@Fe_3_O_4_ core-shell NPs with high *M_s_*and enhanced SAR, in which Fe_3_O_4_ shell protected the Fe core from being oxidized by air to achieve better stability in ambient condition 63. For ferrite-based MNPs, the occupied-position of metal atoms in the octahedral or tetrahedral sites can affect their magnetic properties. The amounts of divalent transition metal cations (*e.g.*, Fe^2+^, Mn^2+^, Ni^2+^, Co^2+,^ or Zn^2+^) can be doped to adjust the *M_s_* and to improve the SAR value further 22, 24, 64-66. It was demonstrated in the report of the higher SAR exhibited by MnFe_2_O_4_ NPs with higher *M_s_* 64 as compared to the MIONs 26. Apart from* M_s_*, *K* can also be controlled by changing the chemical composition of MNPs. Recently, Jang *et al.* 24 developed biocompatible Mg shallow doped γ-Fe_2_O_3_ (Mg_x_-γ-Fe_2_O_3_), with ≈7 nm core size of Mg_x_-γ-Fe_2_O_3_ MNPs (x = 0, 0.05, 0.13, 0.10, and 0.15) (Figure 3a). By controlling Mg^2+^ ion doping amounts in γ-Fe_2_O_3_, the as-synthesized Mg_0.13_@γ-Fe_2_O_3_ exhibited improved magnetic susceptibility which was related with faster relaxation time or magnetic hysteresis loss. Hence, Mg_0.13_@γ-Fe_2_O_3_ exhibited exceptionally high ILP of 14 nH m^2^ kg^-1^, which was two orders of magnitude higher than that of commercial MIONs (Feridex, ILP = 0.15 nH m^2^ kg^-1^) (Figure 3b). The *in vivo* MH studies were carried out with Hep3B xenografted animal models to examine its efficacy using Mg_0.13_@γ-Fe_2_O_3_ nanofluids in cancer treatment. The tumor was completely necrotized from 2 d by MH using Mg_0.13_@γ-Fe_2_O_3_ nanofluids (Figure 3c-d).

**Figure 4:**
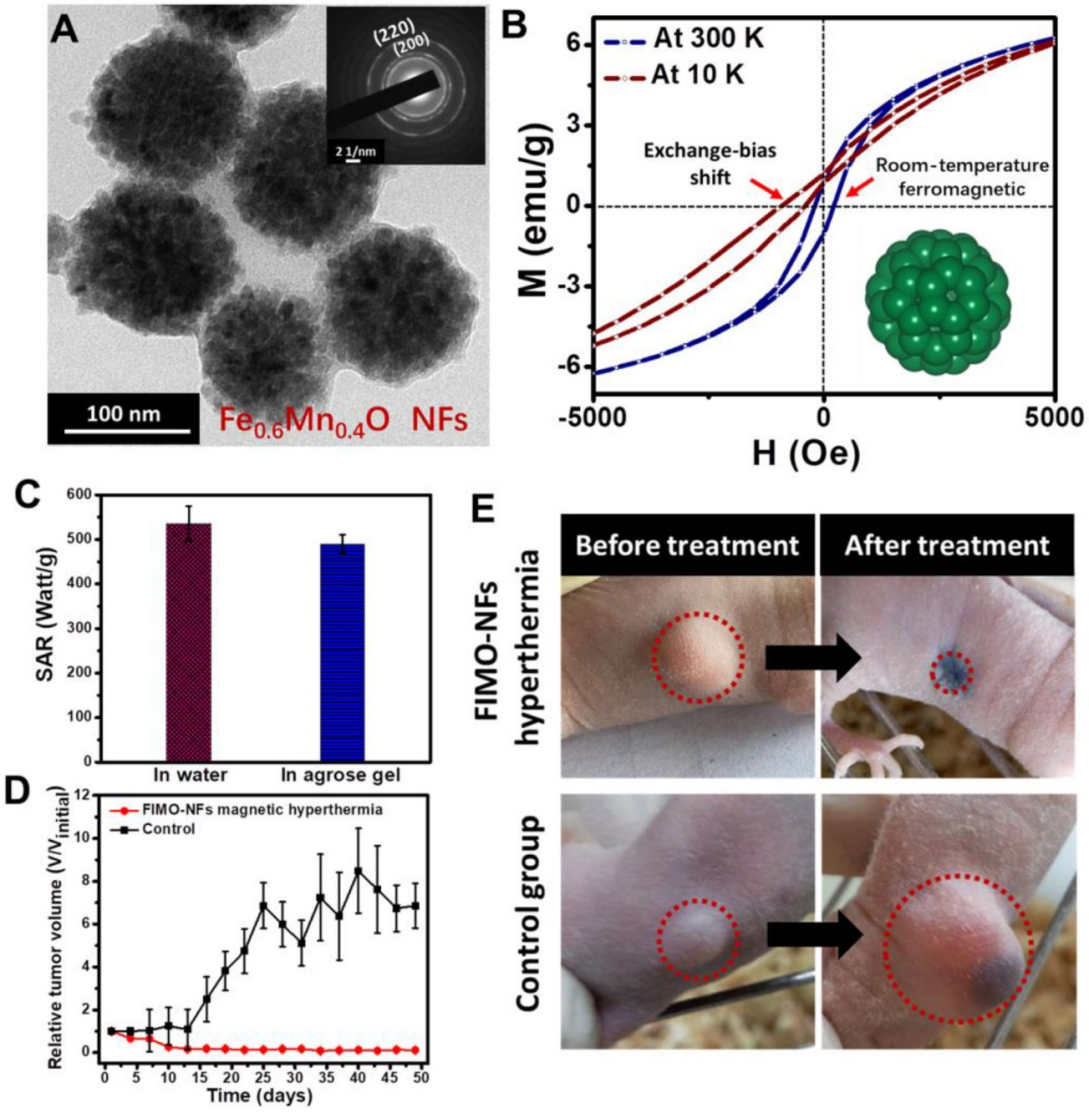
** A.** TEM image of FIMO-NFs, insert showing the SAED image of FIMO-NFs. **B.** Hysteresis loops of FIMO-NFs at 300 K and 10 K. **C.** Comparison of SAR value for FIMO-NFs dispersed in 5% agarose gel and in water. **D.** Tumor growth curves of FIMO-NFs MH group of tumor-bearing mice after treatment and control group. The tumor volumes were normalized to their initial sizes. Error bars represent the standard deviations of three mice per group. **E.** Photographs at day 50 of representative mice from two groups: FIMO-NFs MH treatment group and control group. Reproduced with permission from ref 21. Copyright 2016 John Wiley & Sons Ltd.

Further improvement of SAR has been achieved by enhancing *K* through exchange-coupling. Typically, Lee *et al.* 20 made use of an exchange-coupling between a magnetically soft shell and magnetically hard core to adjust the magnetic properties of the MNPs and to maximize the magnetic loss. The optimized CoFe_2_O_4_@Zn_0.4_Fe_2.6_O_4_ exhibited extremely higher SLP values that are in orders of magnitude higher than conventional MIONs. It is worth noting that the as-reported non-superparamagnetic MNPs exhibited remanence/coercivity, which may bring undesired agglomeration. The presence of other metal elements, such as Co and Ni in these MNPs, may raise concerns regarding toxicity. Afterward, Liu *et al.* 21 fabricated wüstite Fe_0.6_Mn_0.4_O nanoflowers (NFs) with a diameter of 102.7 nm (Figure 4a). By controlling the composition, the obtained Fe_0.6_Mn_0.4_O NFs showed room-temperature ferromagnetic behavior at 300 K, which was different from their antiferromagnetic bulk counterpart. Due to the exchange coupling between antiferromagnet and ferromagnet, exchange bias was generated (Figure 4b). The as-synthesized Fe_0.6_Mn_0.4_O NFs exhibited excellent magnetic induction heating effects, and from the demonstration of the *in vivo* MNPs-MH antitumor experiment, it was shown that Fe_0.6_Mn_0.4_O NFs-mediated MH could induce tumor inhibition effects (Figure 4c-e).

**Figure 5:**
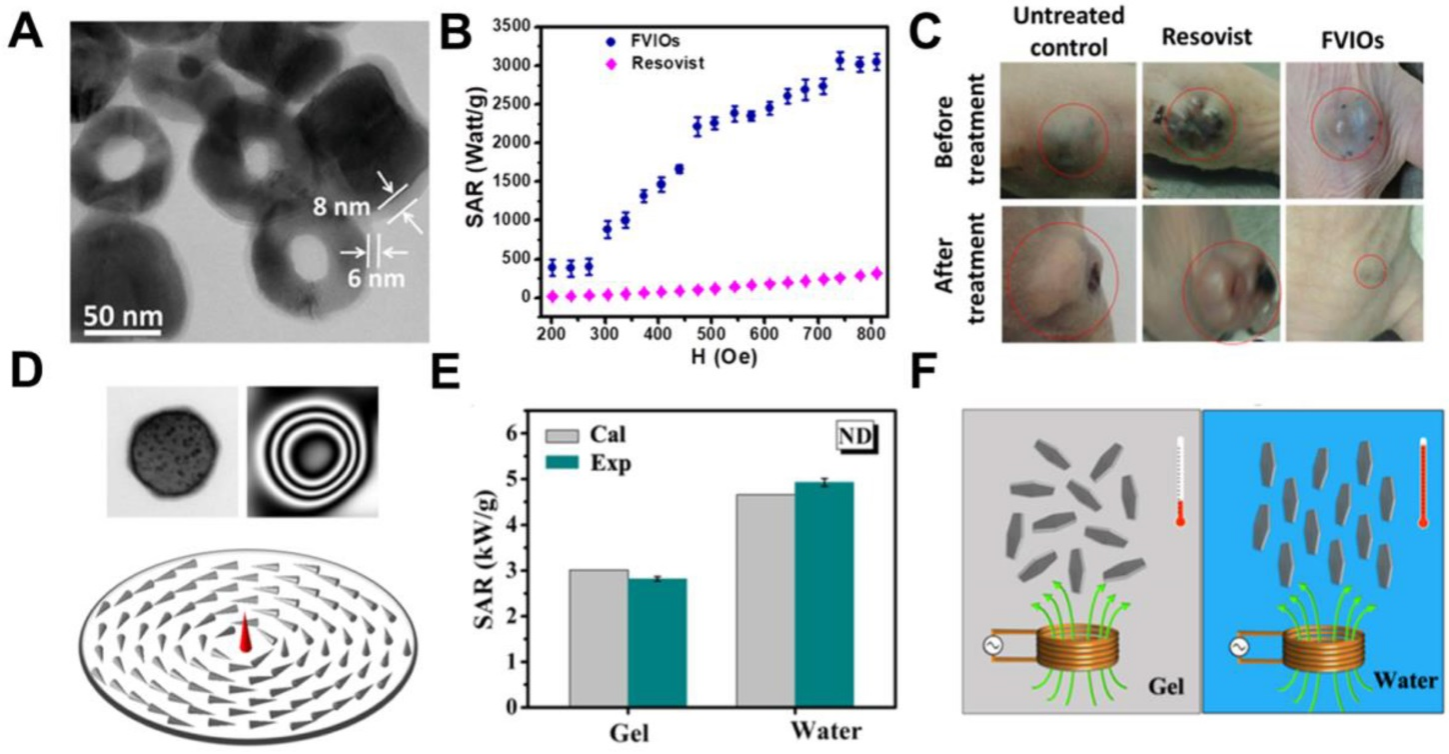
**A.** TEM image of FVIOs. **B.** Comparison of SAR for FVIOs and Resovist in different fields. The frequency is 400 kHz. **C.** Nude mice xenografted with breast cancer cells (MCF-7) before treatment (upper row, dotted circle) and 40 days after treatment (lower row) with untreated control, Resovist hyperthermia, and FVIOs hyperthermia, respectively. Reproduced with permission from ref 31. Copyright 2015 John Wiley & Sons Ltd. **D.** TEM (upper left), electron holography patterns (upper right), and simulated magnetic ground state of a single Fe_3_O_4_ nanodisc (bottom). **E.** Comparison between experimental and calculated SAR values in gel and aqueous suspension when subjected to AMF with 47.8 kA m^-1^ and 488 kHz. **F.** Illustration of orientations related MH of nanodisc in different suspensions. Reproduced with permission from ref 29. Copyright 2014 John Wiley & Sons Ltd.

In addition to the composition regulation, shape is another optimization factor for achieving high SAR. Different morphology causes diverse shape *K*, leading to an extraordinary difference in magnetic loss 28, 73-75. For instance, Serantes *et al.* observed that chain-like magnetotactic bacteria possessed better heating performance than the random orientation system, which indicates the importance of shape *K* 76. Gandia *et al.* [75]also prove that magnetotactic bacteria of the species *M. gryphiswaldense* were very promising as MH agents for cancer treatment. It was demonstrated that the biological structure of the magnetosome chain of magnetotactic bacteria is perfect to enhance the hyperthermia efficiency. Till now, there are conventional MNPs designed with unique morphologies to improve SAR with certain experimental conditions, mainly cubic 28, 77, 78, ring-shape 31, and nanodisc-shape 29. Noh *et al.* reported that Zn_0.4_Fe_2.6_O_4_ nanocubes exhibited significantly higher SAR than that of spherical Zn_0.4_Fe_2.6_O_4_ NPs, despite their identical size and composition. According to the simulation results on the surface magnetic rotation structure, surface disordered spins for cubic Zn_0.4_Fe_2.6_O_4_ NPs were found to be 4%, which was lower than that of spherical Zn_0.4_Fe_2.6_O_4_ NPs (8%) and therefore leading to a high *M_s_* (165 emu/g) and enhanced SAR for the cubic Zn_0.4_Fe_2.6_O_4_ NPs 28. Similarly, cubic morphology was further validated by Martinez-Boubeta *et al.* 79 whereby it was shown through both experimentally and using Monte Carlo simulation that cubic MIONs exhibited higher SAR than spherical MIONs. It has been reported that MIONs with magnetic vortex structure could also improve the SAR 80, 81. Liu and colleagues 31 designed ferrimagnetic vortex-domain nanorings (FVIOs) with an outer diameter of 70 nm (Figure 5a). These FVIOs possessed a much higher *M_s_* and large hysteresis loop that is excellent for large magnetic heat induction. When exposed to an external AMF, FVIOs will transform from a vortex state to an onion state. This transformation leads to a significantly enhanced magneto-thermal conversion ability, with the highest SAR value up to 3050 W/g Fe (400 kHz, 740 Oe), which was one order of magnitude higher than that of commercial MIONs (ferumoxytol, ∼250 W/g) (Figure 5b). Based on the* in vivo* anti-tumor experiments, significant antitumor efficacy by FVIOs on tumor- bearing nude mice was demonstrated (Figure 5c). The high SAR value of FVIOs allowed a very low dosage of 0.3 mg/cm^3^ to be injected into the tumor, which is less than that of reported studies (about 5-10 mg/cm^3^ of tumor tissue). Similarly, Yang *et al.* 29 observed ring-like magnetic flux and demonstrated a vortex domain structure in the MIONs nanodisc (Figure 5d). The SAR of MIONs nanodisc in aqueous suspension and in agarose gel (5%) were 4925 W/g and 2818 W/g, respectively, which agreed well with the simulation results (Figure 5e). The high SAR was resulted from the notion that the parallel alignment of nanodiscs results in a much higher SAR value than random distribution (Figure 5f).

A typical MNPs-MH agent is a system that consists of a magnetic core and coating shell as the two key components. Besides controlling the size, composition, and shape of magnetic core, surface modification is also another factor that can influence the performance of MNPs-MH. MNP's Brownian relaxation in different mediums can be affected by surface coating whereby these coating can act as a 'bridge' in heat transfer into the medium. Liu *et al.* 33 reported that different SAR values could be achieved with different surface coating molecular weight (3 different surface coating molecular weight were used in the experiment, namely poly(ethylene glycol) methyl ether 2000 (mPEG2000), mPEG5000, and mPEG20000). With the decrease in the molecular weight of the surface coating from 5000 to 2000, the highest SAR of 930 W/g was achieved for 19 nm Fe_3_O_4_ core, which represented a 2.5 folds increase.

### Eddy current loss

When a conductor is exposed to an AMF, the eddy current is generated due to two possible factors; (1) relative motion of the field source and conductor, or (2) variations of the field with time. The thermal effect of the eddy current is in accordance with Joule's law. It has been commonly accepted that MNPs are capable of thermogenesis in the presence of AMF. Recently, it was reported that in addition to MNPs, other conductive non-magnetic materials also showed a thermogenic effect when exposed to an AMF. In 2016, Wang *et al.* 82 demonstrated that assembled films of Au NPs exhibited magnetothermal effect in the presence of an AMF, at a frequency of several hundreds of kHz. The mechanism lies in eddy-current heating, which roots in the alteration of the collective conductivity of the Au NPs. Lately, Gao *et al.* 83 was the first group to report the thermogenic effect of a clinically used hypertonic saline (HTS), which exhibited several physiological effects under AMF. This MH system has high safety and biodegradability, and can be used for comprehensive treatment after breast cancer surgery. The emergence of these non-magnetic materials with magnetic field responsiveness provides new research dimensions in the development of MH therapy for cancer treatment.

## Effects of local induction heat

Extensive research has focused on the inductive heating effects of many homogeneously dispersed MNP throughout a macroscopic volume. Recent investigations have suggested that the heat generated by a single MNP could be highly localized at nanometer ranges within the surrounding environment of MNP 37. This local-thermia could be utilized as an incentive factor to regulate cell function/intracellular components, which may contribute to cancer cell death. In 1979, Gordon *et al.* 36 demonstrated a more effective cell apoptosis/necrosis inducing effect with internalized MNPs due to the insulated cell membrane, which enhanced the thermal effect. Sanz *et al.* 38 reported MNPs-MH enhanced cell toxicity regarding exogenous heating, which fully proved that intracellular hyperthermia is possible.

**Figure 6:**
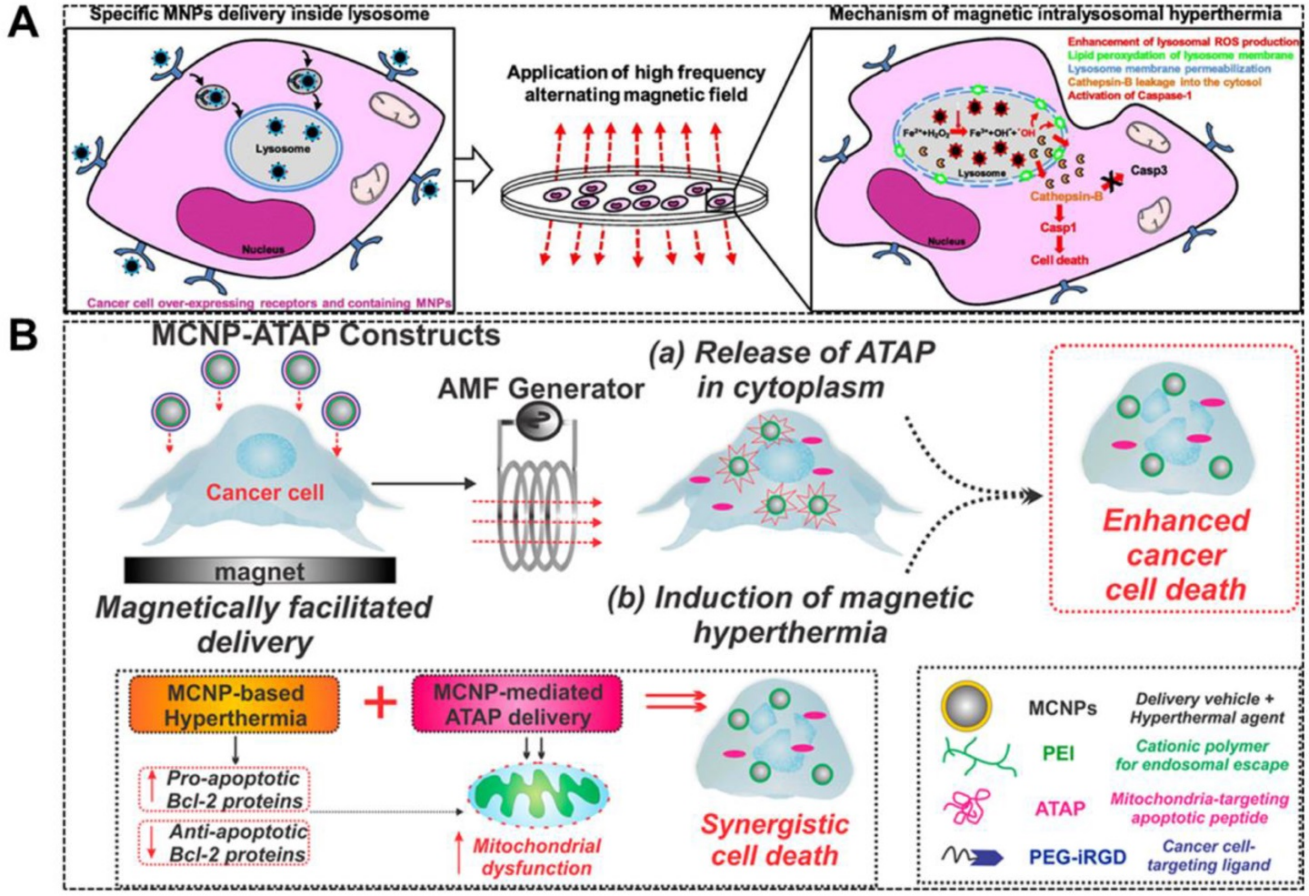
**A.** Schematic diagram depicting magnetically facilitated targeted delivery of MCNP-ATAP to brain and breast cancer cells, wherein the release of ATAP from the MCNPs in the cytoplasm coupled with induction of magnetic hyperthermia in the presence of an AMF can result in synergistic cell death. Reproduced with permission from ref 85. Copyright 2014 American Chemical Society. **B.** Schematic representation of MILH approach. Gastrin-MNPs are specifically internalized through a CCK2R-dependent physiological process, and then they are trafficked to lysosomes where they accumulated. Upon AMF exposure, Gastrin-MNPs cause LMP and the leakage of lysosomal enzymes into the cytosol including Cathepsins which trigger cell death. Reproduced with permission from ref 39. Copyright 2017 Elsevier

With the development of nanotechnology and the ability of targeting MNPs to specific subcellular structures in cells, it is more attractive to use MNPs-MH due to the selective destruction of intracellular targets. The increase in subcellular temperatures, such as focally localized within lysosomes, might induce cell death through lysosomal death pathways, and thus is likely to be applicable to apoptosis-resistant cancer cells. In 2011, *Carlos Rinaldi* and colleagues 84 demonstrated that EGFR-targeted MNPs induced local heat was able to kill cancer cells without detectable macroscopic temperature rise. It was speculated that the possible mechanism was the thermal/mechanical actuation of the apoptotic pathway of EGFR. Domenech *et al.* 40 further demonstrated the potential mechanism. It was observed that upon AMF exposure, EGFR-targeted MNPs were accumulated in the lysosomes of cells and disrupted lysosomes, which led to an enormous reduction in cell viability and an increase in ROS production. Throughout the experiment, the sample temperature remained at 37 ºC. Besides targeting on the disruption of lysosomes, mitochondria-targeting MNPs with localized hyperthermia is also reported to enhance apoptosis in cancer cells. Shah *et al.* 85 reported a magnetic core-shell nanoparticle (MCNP) that can deliver a mitochondria-targeting pro-apoptotic amphipathic tail-anchoring peptide (ATAP) to cancer cell (Figure 6a). MCNP-based targeted delivery of ATAP combined with localized thermal effect resulted in a significantly enhanced apoptosis due to their synergism on mitochondrial dysfunction in cancer cells.

It has been previously reported that the local heating occurs near the surface of MNPs can induce biological effects and regulate the physiological and biochemical properties of certain molecules, and thus causing functional changes in the whole organism. Huang and colleagues demonstrated that MNPs targeting the outer cell membrane could activate ions channels with a very focal temperature increase without thermally affecting the Golgi apparatus 34. It is well accepted that Fe_3_O_4_ nanoparticles are classical Fenton nanoagents which can generate ROS 86, and hence able to induce tumor cell apoptosis. It is noteworthy that the active MIONs under AMF can amplify the ROS generation in tumor microenvironments (TME), leading to an enhanced therapeutic effect 87. Clerc *et al.* 39 used magnetic intra-lysosomal hyperthermia (MILH) to prove that cell death through a non-apoptotic signaling pathway can be caused by using Gastrin-grafted MNPs specifically delivered to lysosomes of the tumor cells (Figure 6b). The cell death was attributed to the local temperature increase at the periphery of the MNPs which enhances the ROS production through lysosomal Fenton reaction. After which, MILH induces lipid peroxidation, lysosomal membrane permeabilization, and leakage of lysosomal enzymes into the cytosol, including Cathepsin-B which activates Caspase-1 but not apoptotic Caspase-3. This work elucidated the cellular and molecular mechanisms involved in cancer cell death induced by MILH.

**Figure 7:**
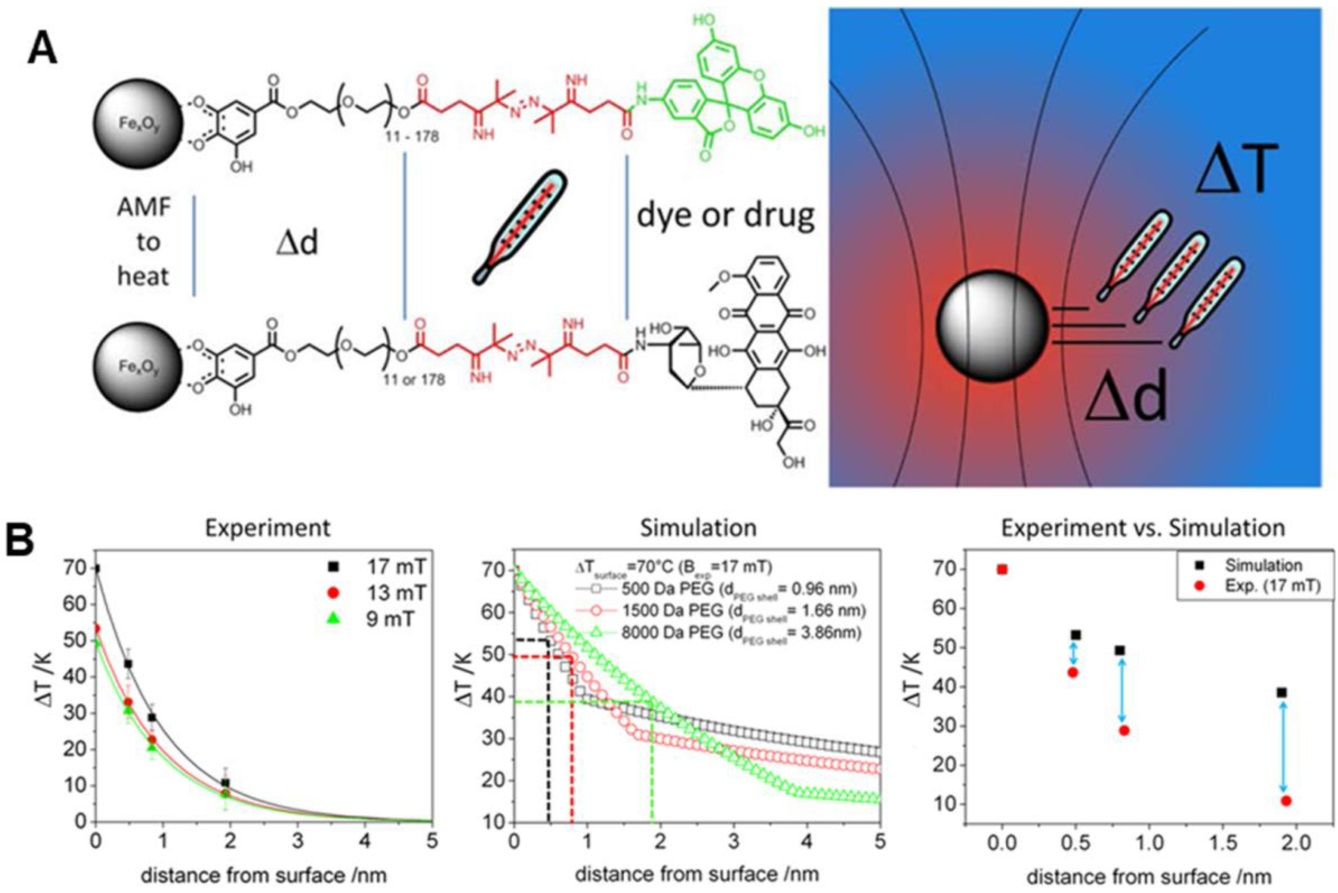
** A.** Schematic representation of measuring the temperature profile at the nanoparticle surface with a subnanometer resolution. **B.** Experimental temperature gradients for all field amplitudes, temperature gradients calculated by applying the Fourier law for three different shell thicknesses (PEG500, PEG1500, and PEG8000) and comparison of ΔT values at 0, 0.47, 0.83, and 1.9 nm. Reproduced with permission from ref 52. Copyright 2013 American Chemical Society.

The generation and the measurement of local temperature in the nanometer range within the surrounding environment of MNP is an important aspect in the biological system, and also for MNPs that are used in inducing hyperthermia, heat stimulation, and drug release 8, 34, 88-90. Researchers during *in vivo* or *in vitro* experiments are able to measure only the bulk or macroscopic temperatures in the system. However, the measurement of temperature at the surface of MNPs within the nano-scale spatial resolution is of pivotal importance in deciding the effectiveness and the minimum required MNPs concentration to achieve the objective without any side effects. Furthermore, the bulk temperature around the MNPs is entirely different from the surface temperature. As such, during the activation of temperature-sensitive TRPV1 protein channels to influx calcium ions at 40°C using MNPs, no change in bulk temperature was observed even with the use of optical fiber 34. Research groups 71-75 have been working on the measurement of the surface/local temperature of MNPs using thermally active bio-indicators. Riedinger *et al.* 52 fabricated 15 nm MIONs, modified with polyethylene glycol (PEG) oligomer terminated by a fluoresceine-amine dye attached to a thermolabile azo linker (Figure 7a). The molecular weight of the PEG was modified from 500 Da to 1500 Da and 8000 Da. These molecular weights created difference separations of 0.47 nm, 0.83 nm, and 1.9 nm between the bulk to the surface of the nanoparticle, with measured temperature increments of 42 °C, 26 °C, and 11 °C, respectively (Figure 7b). It was discovered that significant local heating was achieved at distances below 0.5 nm with an increased temperature of up to 45 °C, which decreased exponentially with increasing distance. Furthermore, the temperature increase was found to scale linearly with the applied field at all distances 91. Hence, a molecular spacer based temperature-sensitive three-component system, MNPs-A-B-C, could be proposed for detecting the temperature at the surface of MNPs within a nano-scale spatial resolution. “A” is a variable molecule with different molecular weight to vary the thickness of the shell, “B” is the thermolabile molecule and “C” is the fluorescent indicator which gets activated when released in the aqueous system. The decomposition rate calculated by fluorescent intensity gives the local temperature and its gradient.

Localized heat has unique advantages in regulating biological molecules: (1) It is directional, efficient, and safe as it can only affect macromolecules within 10 nm around MNPs, and the hot spot temperature is extremely high. (2) The generation of localized heat is instantaneous and can be used as a "molecular switch" to regulate the function of the interaction molecules, so as to achieve the goal of disease treatment. Accordingly, the biological effect of localized induction heat can be redefined with the following characteristics: (1) no macro-heat is produced, (2) under AMF, MIONs can effectively regulate the interaction between specific macromolecules or change the TME to achieve selective tumor cells killing effect, and (3) heat can achieve extremely selective and targeted therapies.

### MNPs-MH therapy-based synergistic strategy

In addition to primarily using MNPs-MH therapy to kill the tumor cells *via* inductive heating, it can be used as an adjuvant treatment to clinical radiotherapy and chemotherapy, as well as to be used in synergism with immunotherapy, and photothermal/PDT *et al*. It has great significance in reducing its toxicity and side effects, thus improving the prognosis of patients. In addition to a cumulative effect, a much sought-after syngersim can be achieved by the mutual enhancement effect of the individual cytotoxic mechanism of each strategy on each other.

### MNPs-MH combined with chemotherapy

MIONs not only can be utilized for targeted drug delivery and controlled drug release under an external field, but it can also be used as a trigger for magnetic field-mediated hyperthermia synergistic chemotherapy. Numerous studies have shown that MNPs-MH can induce vasodilation, *i.e.*, the expansion of tumor blood vessels, which results in increased blood circulation and, therefore, effectively accelerating the intracellular drug delivery and release. Also, MNPs-MH provides a thermal enhancement that improves the drug cytotoxicity that is accompanied by interfering with the repair mechanism of tumor DNA and expression of multidrug resistance P-glycoprotein, thus damaging the way of tumor cells resisting apoptosis 92, 93.

MNPs-MH helps in the increased intracellular uptake of drugs by changing the permeability of the cell membrane. Berríos *et al*. 94 measured the cell membrane fluidity with some direct and indirect methods. They provided persuasive proof that MH can induce significantly greater cell membrane permeability as compared to hot water hyperthermia at similar temperature conditions. Inspired by heat-induced changes in the cell membrane permeability, Tabatabaei *et al.* 95 demonstrated that the MNPs in regulated radio frequency (RF) field could act as miniaturized heat sources which transfer the thermal energy exclusively to the endothelium with higher spatial precision. Results indicated a substantial but reversible opening of the blood-brain barrier (BBB) where MH is applied.

**Figure 8:**
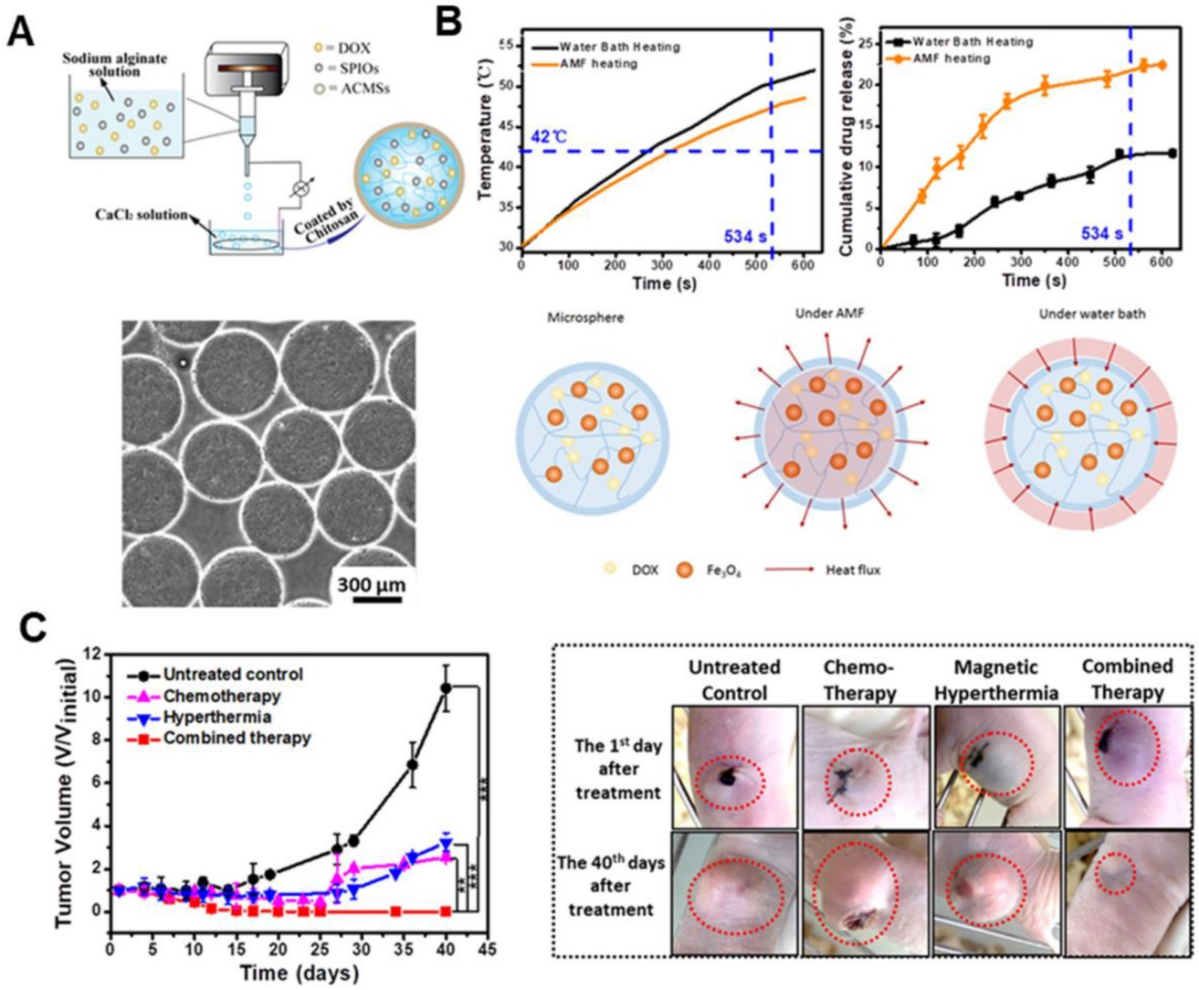
** A.** Schematic diagram of a facile electrostatic droplet generation technology to prepare DM-ACMSs (top), and the optical micrographs of 510 µm DM-ACMSs with a Fe concentration of 0.29 mg mL^-1^ ACMSs (bottom). **B.** T-t plot (upper left) and cumulative release profile of DOX (upper right) for DM-ACMSs-0.29 under an AMF (40 kA/m; 265 kHz) or by water bath heating, and schematic illustration of the heat transfer process of a DM-ACMS under the AMF and water bath (bottom). **C.** Time-dependent changes in tumor volume for mice with different treatments. Reproduced with permission from ref 102. Copyright 2018 Royal Society of Chemistry.

MIONs can be used as a nanocarrier for encapsulating drugs to enhance chemotherapy upon AMF exposure. Furthermore, by facilely regulating the AMF frequency, both the heat generation and drug release can be controlled for on-demand synergistic MH/chemotherapy 96-98. Zhou *et al*. 99 designed a core-shell Fe_2_O_3_@PPy-DOX-PEG nanocomposite. The release of DOX from the nanocomposite can be set off by acid stimulus and AMF, which resulted in an outstanding combinatorial therapeutic effect. Ullah *et al*. 100 reported a macrophage-based drug delivery system with MH controlled drug release system using a thermolabile linker. The release of the toxin could specifically induce > 70% death of Kaposi's sarcoma cells in a 3D spheroid co-culture even at a low ratio of 1:40 (SPION loaded macrophage: tumor cells). The spatio-temporal drug delivery and release achieved by regulating the AMF frequency is highly desired for realizing on- demand synergistic MH/chemotherapy. This helps in achieving optimal treatment efficacy with minimal side effects as compared to the MH-accelerated drug release. Jia *et al.* 101 designed a glioma-targeting exosome, which carried SPION and Curcumin. It could cross the BBB smoothly and provided good results for targeted imaging and potent synergistic antitumor effect between SPION-mediated MH and Cur-mediated therapy. Furthermore, Xue *et al*. 102 designed magnetic hydrogel microspheres, which composed of DOX, MIONs, and alginate/chitosan microspheres (DM-ACMSs), as a model system to provide insight into the underlying mechanism of AMF stimulated drug release behavior in non-thermal sensitive hydrogel system. DM-ACMSs with controllable size were prepared by a facile electrostatic droplet generation technology (Figure 8a). Under a remote AMF, DM-ACMs rapidly reached a 22.5% cumulative drug release within 10 min upon exposure under AMF. On the other hand, only 0.2% DOX was released in the absence of AMF (Figure 8b). Moreover, the cumulative drug release amount under AMF is approximately two times higher than that by water bath heating. Further analysis revealed that the AMF stimulated drug release was driven by both thermal and concentration gradient from inside to outside. It can be well-described by the mass and heat transfer coupling mechanism using the Soret diffusion. The *in vivo* anti-tumor effect on tumor-bearing mice revealed the disappearance of residual tumors in 12 days after chemo-thermal synergistic treatment using DM- ACMSs (Figure 8c).

### MNPs-MH combined with radiotherapy

Since the discovery of X-rays by Roentgen, radiation therapy (RT) has been portrayed as a standard treatment modality for cancer. However, the therapeutic effect is usually masked by its side effects faced by the normal tissue and the radiation resistance induced by hypoxia. MH plays a crucial role in the process of radiosensitization, which can enhance the damage to the tumor cells and blood vessels by interfering with the repair mechanism of tumor DNA after their injury. It has been demonstrated that the combination of MNPs-MH and RT not only can effectively kill resistant cells and lower toxicity to normal tissue, it can also lower the radiation dosage 103, 104. Jiang *et al*. 105 designed a gadolinium-doped iron oxide nanoparticles (GdIONP) with higher SAR, and they explored its therapeutic effects when used in the combinatorial radio-thermotherapy. The results indicated that the efficacy of RT could be enhanced with GdIONP-mediated hyperthermia in two ways; (1) by reducing the fraction of hypoxic cells that contribute to radiation resistance and, (2) by inducing tumor-specific localized vascular disruption and necrosis. Due to the difficulty of external energy sources in reaching the targets to generate adequate heat, significant benefits of thermo-radiotherapy may be limited to superficial tumors 106. Nevertheless, MH can also act as an adjuvant treatment to radiotherapy for metastatic breast cancer. Wang *et al.* 107 revealed a three synergistic actions by MH that resulted in a tremendous improvement in lung metastasis and in overall survival of mice under combinatorial MH/RT treatment; (1) promoting the anti-tumor efficiency of radiotherapy through Bax-mediated cell death, (2) improving cellular immunity which is suppressed under radiotherapy, and (3) decreasing the potential of radiotherapy to enhance MMP-9 expression.

MIONs can also increase the efficacy of RT with the help of ROS. RT promotes mitochondrial respiration and therefore increases the production of superoxide anion, which is further converted to hydrogen peroxide. Subsequently, the formation of highly reactive hydroxyl radicals is catalyzed by the MIONs, which results in enhanced toxicity. Hauser *et al*. 108 reported that TAT functionalized nanoparticles combined with radiation resulted in synergistic cytotoxic effects on A549 lung carcinoma due to the increased ROS generation.

### MNPs-MH combined with immunotherapy

In addition to the killing of cancer cells with heat, MNPs-MH can also trigger an anti-tumor immune response by releasing tumor antigens and endogenous adjuvants (*e.g.*, heat shock proteins and damage-associated molecular patterns), which demonstrates significant potential for tumor therapy (especially for metastatic tumors) 109-111. In 1998, Kobayashi *et al.* carried out an experiment whereby the left side of the tumor was subjected to MNPs-MH treatment mediated by magnetite liposomes. By transplanting a T-9 rat glioma tumor model into each femur of a rat, the distant tumor also showed inhibited progression even without subjected to hyperthermia. It was revealed that CD3^+^, CD4^+^, CD8^+^, and NK cells were detected in both the left and right tumor tissues of the rats, which lead to long-lasting and T-9 cell-specific acquired immunity 112.

**Figure 9:**
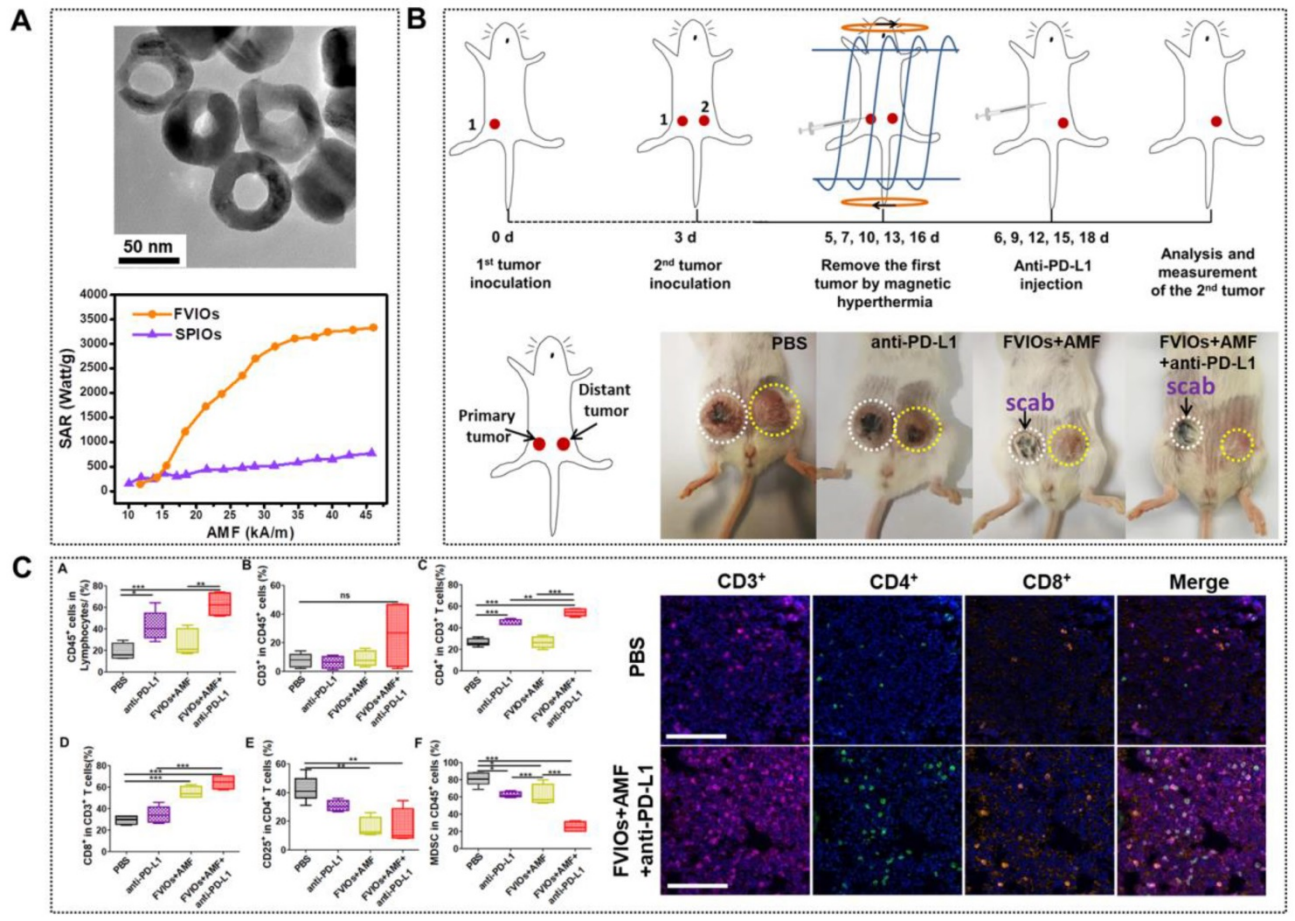
** A.** TEM of PEGylated FVIOs (top) and SAR comparison of PEGylated FVIOs and SPIONs at different fields (bottom). **B.** Schematic illustration of the experimental design and photographs of mice after treatments. **C.** FVIO-mediated mild magnetic hyperthermia therapy plus immune checkpoint therapy activates the antitumor immune response (left) and representative multispectral fluorescence images of distant tumors after immunofluorescence staining (right, scale bar = 100 μm). Reproduced with permission from ref 113. Copyright 2019 American Chemical Society.

In spite of all these progressions, tumor recurrence and metastasis remain after MNPs-MH treatment. Recent studies showed that immune therapies could prevent the tumor from recurrence and metastasis by stimulating and activating the antitumor immune system to attack tumor cells specifically. However, the durable response produced by immune therapy is too low. Recently, it was reported that MNPs-MH, when used with immune checkpoint blockade, could result in systemic therapeutic responses to inhibit tumor metastasis. Liu *et al*. 113 reported an FVIO-mediated mild MH with anti-PD-L1 treatment for the orthotopic 4T1 tumor model locally treated with AMF, with resulting eradication of the primary tumors. This prevented lung metastases and also inhibited the growth of distant tumors (Figure 9a-b). They demonstrated that the MH could up-regulate CD8^+^ cytotoxic T lymphocyte infiltration in distant tumors, which effectively sensitized tumors to the PD-L1 checkpoint blockade. Meanwhile, the combinatorial approach also down-regulated myeloid-derived suppressor cells (MDSCs) (Figure 9c). Chao *et al*. 114 further demonstrated that the combination of FeNP-based MH with local injection of nanoadjuvant and systemic injection of anti-cytotoxic T lymphocyte antigen-4 checkpoint blockade could inhibit tumor metastasis.

### MNPs-MH combined with PTT or PDT

Although MNPs-MH has been in clinical trials as an effective cancer treatment, there are certain drawbacks that need to be addressed. Some of the drawbacks that limit the clinical applications of MNPs-MH include the requirement of high MNPs concentration and the significant reduction of heating efficiency in the cellular environment. Moreover, due to the low heating efficiency of the MNPs, MNPs-MH can only be performed through direct intratumoral injection, which significantly limits the realization of precise MNPs-MH owing to the inhomogeneous distribution of MNPs.

**Figure 10:**
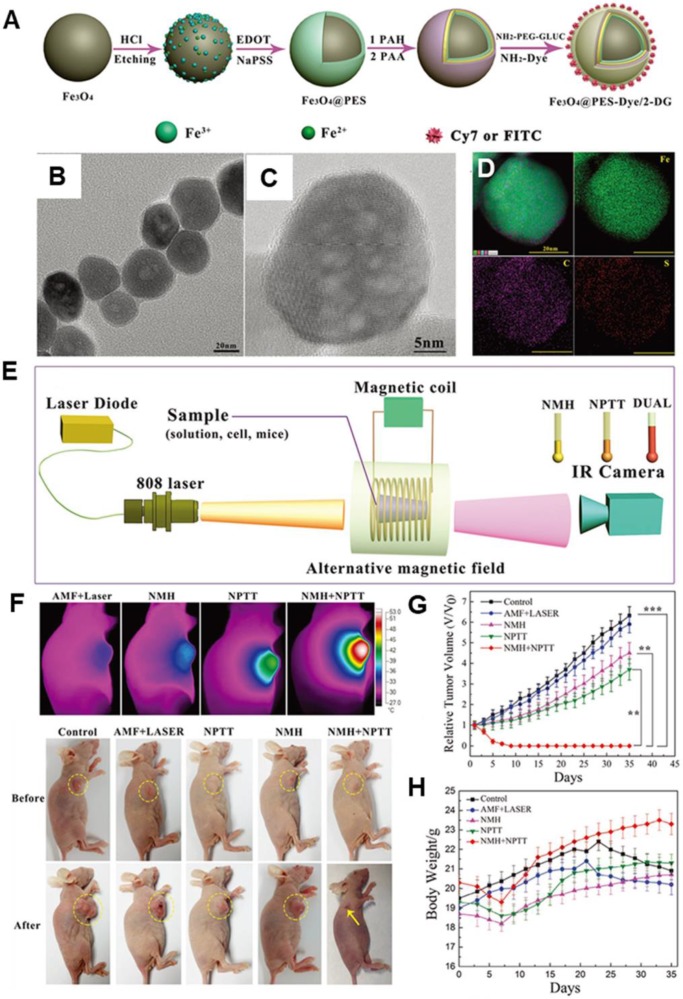
**A.** A schematic diagram of the MNP@PES-Cy7/2-DG fabrication process. **B.** TEM image of the MNP@PEDOT:PSS nanocomposite. **C.** High-resolution TEM image.** D.** EDS mapping images, scale bar represents 20 nm. **E.** Scheme of the experimental device for combined hyperthermia experiments. **F.** IR thermal images of groups under four different heating protocols and representative images of different groups before and 35 d after treatment. **G.** Growth of MCF-7 tumors in different groups of mice after various treatments (tumor volumes were normalized against their initial sizes). Error bars represent standard deviations. **H.** Body weights of mice in different groups after various treatments. Reproduced with permission from ref 119. Copyright 2018 John Wiley & Sons Ltd.

Nano-photothermal therapy (NPTT) is another popular and developed nanotechnology for cancer therapy. However, drawbacks such as high laser irradiation dosage and inevitable depth-dependent decline of laser intensity have significantly curbed the clinic translation for NPTT. It has been demonstrated that the combination of MNPs-MH and NPTT not only can provide the cumulative heat effect, but it can also achieve a synergic effect of 1+1>2 115-118. Yan* et al.* 119 designed a multifunctional theranostic nanoplatform that was composed of MIONs, Cyanine7 (Cy7), poly(3,4-ethylenedioxythiophene): poly (4-styrenesulfonate) (PES), and 2-deoxyglucose (2-DG)-polyethylene glycol to achieve *in vivo* photo-magnetic hyperthermia. The nanoparticles (30-40 nm in size) (Figure 10a-d) were aggregated mainly in the cytoplasm of tumor cells *in vitro* and *in vivo*, and they exhibited stealth-like behavior with a long second-phase blood circulation half-life of 20.38 ± 4.18 h. Under the simultaneous guidance imaging, those multifunctional nanoprobes were delivered to the breast tumor site by intravenous injection for additive photo-magnetic hyperthermia (Figure 10e-h). The results showed that the temperature in the tumor increased by 22°C during photomagnetic hyperthermia treatment, which was consistent with the additive effect of the combined *in vitro* hyperthermia. Recently*,* Ma *et al*. 87 designed a biocompatible Fe_3_O_4_-Pd Janus NPs that were able to achieve significantly higher magnetic-photo heating efficiency that was accompanied with enhanced ROS generation. As such, a high tumor-inhibition efficacy was exhibited towards 4T1 orthotopic breast tumors with a 100% inhibition rate in an animal model.

In addition to combining with NPTT, combined MNPs-MH/PDT is also promising in improving the antitumor efficacy. PDT is a powerful technique used to activate malignant cells apoptosis photochemically, which has shown potential in many clinical applications. However, due to the transport limitation of therapeutics and the oxygen-dependent nature of PDT, malignant cells in hypoxic regions are often resistant to PDT. Huang *et al*. 120 successfully developed an oxygen/therapeutic payload delivery technique to effectively enhance the antitumor efficacy, as well as to overcome the limitation of PDT in tumor hypoxia. Bone marrow-derived monocytes were explored as cellular vehicles for co-transporting of oxygen and photosensitizer, chlorin e6, and SPIONs. Significant apoptosis of cancer cells co-incubated with therapeutic monocytes occurred by virtue of the combinatorial effect of PDT and MNPs-MH, under AMF treatment and red light laser irradiation. Curcio *et al*. 121 designed an all-in-one nanohybrids merging MH, PTT, and PDT in a tri-therapeutic strategy by combining a nanoflower-like γ-Fe_2_O_3_ core optimized for MH, and a CuS spiky shell honed for IR-triggered PTT and PDT. The combinatorial treatment approach with MH and PTT can provide a cumulative heating effect enabling lesser dosage and greater effectiveness.

### MNPs-MH combined with gene therapy

MNPs-MH can lead to cancer cell death via the localized heat, which makes it an important cancer treatment modality. Gene therapy is another exciting research topic in current cancer treatment. Improving the efficacy can be achieved by controlling gene expression through genetic editing. Cellular systems have developed mechanisms to aid in adapting to thermal stress during evolution by inducing heat-shock proteins (HSPs) as the major target proteins 122. Due to their high conservation across prokaryotes and eukaryotes, their importance in the cellular protection mechanisms is undeniable. By activating compensatory mechanisms by the heat-shock response, the cells are only then able to survive hyperthermia. It was shown that the mechanisms mediating heat-shock response are not strictly for protection towards heat, they also demonstrate protection towards other stress factors. The survivability of the cells under heat-shock is highly dependent on the conditions of the applied heat. This can lead to the therapeutic decision to use a desirable clinical setting in hyperthermia to trigger the cells into apoptotic/necrotic pathways. After heat-shock, the changes in gene expression (numerous genes are up- or down-regulated by heat-shock) are induced shortly and can last into the period of normothermia. HSPs are the major group of proteins expressed by this mechanism, which prevents the misaggregation of denatured protein after stress. In addition, HSPs also involved in the proper folding of nascent proteins into their required functional conformation. These proteins with functional conformation further regulate both the protein turnover and cellular redox-state. Members of the HSP40, HSP60, HSP70, and HSP90 families are some of the most well-known HSPs. The mechanism of their heat-responsive gene expression is of particular interest to the design of heat-responsive gene therapy vectors. Ito *et al.* proposed to enhance the heat stress gene to improve MH effect and to minimize the side effects 123. Stem cell-based gene therapy shows tremendous potential for cancer treatment, but it is hard to control, which can lead to side effects. Yin *et al.* 124 reported magnetic core-shell nanoparticles to deliver and activate a heat-inducible gene vector that encodes tumor necrosis factor (TNF)-related apoptosis-inducing ligand (TRAIL) in adipose-derived mesenchymal stem cells (AD-MSCs). When the engineered AD-MSCS was generating heat under AMF exposure, TRAIL was selectively expressed, which induced significant ovarian cancer cell apoptosis and death *in vitro* and *in vivo*. Moreover, the engineered AD-MSCs still demonstrate their native ability to proliferate, differentiate, and target the tumors. MNPs-MH combined with gene therapy can complement their individual advantages to achieve a significant effect in cancer treatment. Wang *et al.* 125 reported a suicide gene as a promising alternative, which can convert a prodrug into a highly toxic drug. However, the drawback of this suicide gene is the difficulty in controlling its expression. MNPs is an excellent material for drug and gene delivery for various reasons: (1) it can be accumulated in the tumor cell, and then release the drug and gene to improve the effectiveness. (2) MNPs can generate heat under AMF exposure. Hence, magnetically targeted and hyperthermia-enhanced suicide gene therapy of hepatocellular carcinoma (HCC) was achieved.

Besides, multi-synergistic therapy is also reported. Wang *et al.* 126 described the use of immunogenic NPs mediated combination of PDT and MNPs-MH to synergistically augment the anti-metastatic efficacy of immunotherapy, which potentially advance the development of combined MNPs-MH, PDT, and checkpoint blockade immunotherapy to combat cancer metastasis. Although combinatorial therapy is promising in the clinic application, a further degree of complexity is reached when designing NPs with multifunctional capabilities that require defined nanoparticle features. Optimizing the NPs design for one modality does not necessarily improve the other. Hence, various aspects that make the application of MNPs-MH optimal in other treatment modalities have to be considered.

## Imaging-assisted targeted MNPs-MH

Besides its use in MNP-MH applications, there is an existing significant amount of background and knowledge on MNPs in magnetic resonance imaging (MRI) applications of routine diagnostic radiology. Imaging-guided cancer treatment empowers the physicians to locate and treat tumors with greater precision and, therefore, minimizing damage to surrounding tissue. Engineered MNPs can realize MRI contrast enhancement and treatment of local hyperthermia generated by absorbing energy from an AMF. When performing MNPs-MH for cancer treatment, the surrounding normal tissue often suffers from collateral heat damage. Thus, to avoid such damage to healthy tissue, a more specific image guidance is needed for more precise treatment of cancer tissue. Thorat *et al*. 127 designed a functionalized superparamagnetic La_0.7_Sr_0.3_MnO_3_ NPs with an oleic acid-PEG polymeric micelle (PM) structure that is loaded with anticancer drug doxorubicin (DOX-conjugated PM@SPMNPs) nanocomposites to serve as an MRI guidance of MNPs-MH and chemotherapeutic drug delivery. The nanocomposites induced hyperthermia led to cancer cell extinction of up to 80% under *in vitro* conditions within 30 min.

Magnetic particle imaging (MPI) is a noninvasive 3D tomographic imaging method with high sensitivity and contrast without ionizing radiation. In addition, it is linearly quantitative at any depth with no view limitations in its non-invasive imaging technique. MPI is, thus, a powerful and emerging platform for magnetic-based theranostics due to its high contrast and high sensitivity imaging combined with precise control and localization of the therapy. Tay *et al.* 128 demonstrated a theranostic platform with quantitative MPI image-guidance for treatment planning and the use of MPI gradients for spatial localization of MNPs-MH to arbitrarily selected regions. Du and colleagues 26 successfully demonstrated a novel strategy for cancer thermotherapy by developing theranostic MNPs, which induced a significant potentiation of magnetic heat that led to a uniform temperature inside the tumor. This work, to the best of our knowledge, is the first example of MNPs optimized for dual-mode MRI/MPI image-guided MNPs-MH *in vivo*. They methodically investigated the effects of diameters and components of MFNPs on the performances of MPI and MRI signal properties. It was discovered that 18 nm Fe_3_O_4_ NPs demonstrated better MPI and MRI signal properties as compared to other developed MNPs that include commercially available Vivotrax. 18 nm Fe_3_O_4_ NPs was designed to leverage their advantages and potential of MRI/MPI dual-mode and MNPs-MH for precision imaging and cancer therapy.

## Clinical Trials of MNPs-MH

Due to all these therapeutic benefits of focal nanothermic action in tumor treatments, MNPs-based therapy is highly attractive 129. NanoTherm®therapy (trade name), the world's first MNP based therapy for prostate and brain tumors, has been extensively studied in preclinical settings and has recently translated into clinical evaluations (clinical trials NCT02033447 (prostate), and DRKS00005476 (glioblastoma) for MNPs-MH therapy. The first prototype of a clinical MNPs-MH therapy system was set up at the end of 2000 at the Charité Medical School, Campus Virchow-Klinikum, Clinic of Radiation Oncology in Berlin. It is a ferrite-core applicator operating at a frequency of 100 kHz with an adjustable vertical aperture of 30 -45 cm. The field strength is adjustable from 0 to 15 kA/m. Two phase-I trials were conducted for MNPs-MH: (1) MNPs-MH in combination with external radiotherapy was used to treat patients suffering from recurrences of glioblastoma multiforme, and (2) thermotherapy alone was administered to patients with recurrent prostate carcinoma 130-134. Between March 2003 to January 2005, the world's first phase I trial was conducted on 14 patients with glioblastoma multiforme (GBM) at the Charit´e-University Medicine Berlin 130, 131, 135. The first treatment system for patients was developed at the Charité -University Medicine Berlin. Both the whole-body magnetic field applicator (MFH 300F) and nanofluid with aminosilan coat (MFL 082AS) were optimized for clinical application. A 3D image-guided intratumoral injection of aminosilane-coated iron oxide nanoparticles was conducted under general anesthesia, and the distribution of MNPs throughout recurrent or nonresected primary tumors was confirmed. Patients received six thermal therapies with a duration of 60-min at weekly intervals using an AMF applicator. The median maximum intratumoral temperature was kept around 44.6 ^o^C. The patients received six treatments, with two treatments per week. This phase I trial showed the feasibility, tolerability, and efficacy of MNPs-MH. With the success of the phase I study, a phase II study was subsequently started in January 2005. The evaluation of the treatment efficacy in 65 patients suffering from recurrences of GBM is still in progress. In 2013, MagForce AG started the post-marketing clinical study in recurrent glioblastoma with NanoTherm® Therapy after receiving approval from the German Federal Institute for Drugs and Medical Devices (BfArM). The trial is an open-label, randomized, and controlled study to determine the efficacy and safety of NanoTherm® monotherapy alone and also when used in combination with radiotherapy versus radiotherapy alone in up to 280 glioblastoma patients. In 2019, MagForce USA, Inc. has successfully completed stage 1 of its clinical study on the focal ablation of intermediate-risk prostate cancer, and the next stage is under preparation.

## Challenges and Perspectives

In this review, the current state-of-the-art strategies for improving the therapeutic efficacy of MNPs-MH are discussed. Significant attention has been focused on the use of MNPs to raise the temperature of tumor tissue to the hyperthermia range in order to enable cancer therapy. The capability to tune the size, composition, and morphology of MNPs allows the optimization of MNPs. Ultimately, this leads to sufficient and effective heating properties, which is considered as a direct way of improving therapeutic efficacy. The recent experimental evidence from literature supports the notion that MNPs can be engineered to cause local thermal effects. A deep understanding of this localized heat leading to the disruption of cellular structures and cell death without the need for a macroscopic temperature increase further supports the efficacy of MNPs in the therapy. By synergistically combining MNPs-MH with other therapeutic strategies, such as chemotherapy, radiotherapy, immunotherapy, and photothermal/PDT, it allows further enhancement of the effectiveness of the antitumor treatment. Despite the recent tremendous progress in MNPs-MH 53, several remaining challenges need to be overcome.

(1) Safety aspect is the utmost important concern when using MNPs. While precise control of the physical parameters (size, components, and shape) of MIONs is essential in improving the energy dissipation rate of MIONs, there is still a significant risk arising from the potential toxicity effects introduced by different MIONs physical properties. This will eventually lead to clinical safety risks. Therefore it is essential to balance both the safety and effectiveness of magnetic nanomediators with the ultimate goal of minimizing toxicity to the human body. In addition to the single modality approach, the synergistic effect of MNPs-MH and other therapeutic modalities is achieved with the nanohybrids design. Significant attention has been focused on the unique designs and remarkable chemical/physical properties of nanohybrids which may pose safety concerns as well. Regardless of the therapeutic therapy modalities, “keeping it simple and safe” is strictly required for a successful bench-to-bedside transition and for clinical applications.

(2) The maximum allowable magnetic field amplitude and frequency for MNPs-MH should be adequately elucidated. To date, the effects of AMF exposure on tissues have been poorly studied. Based on the available literature, it is possible to extrapolate the observations of Atkinson *et al.* and Oleson *et al.* practically to obtain guidelines. The maximum frequency and amplitude *H×f* <5×10^9^ is set as the upper limit by the MNPs-MH research community. More extrapolation must be done cautiously and in consideration of the other parameters that can significantly affect eddy heating in tissues.

(3) The biological effect of MNPs-MH in therapeutic effectiveness should be studied in greater detail. Tumors are considered as a complex and constantly evolving ecosystem, with the TME and its anomalies becoming the primary targets for treatments. MNPs are not only taken up by the tumor cells, but they can also be taken up by T lymphocyte, DC, macrophage, and MDSCs *et al.* in the TME with a high probability of similar cellular uptake. Hence, apart from acting on tumor cells and vasculature, the effect of MNPs-MH on the extracellular matrix, T lymphocyte, DC, macrophage and MDSCs, and its modulation of the immunity-associated cell action in the complex physiological environments and cell microenvironments, should be well investigated.

(4) The mechanism of MNP-MH therapy for tumors is very complex, which involves gene, protein, subcellular organelles, tissue structure, *etc.* Systematic study of different levels of magnetothermic biological effects can lay the foundation for efficient MNPs-MH for tumor therapy.

(5) MNPs-MH has claimed reasonable success in direct injection therapy. However, targeted hyperthermia following intravenous injection is still undergoing arduous development. Further optimization of the specificity and selectivity of MNPs is also required so that they can target specific cellular or subcellular sites *in vitro* and *in vivo*. The success in the establishment of MNPs-MH therapy lies significantly in the integrated approach considering both the physical constraints of the MNPs-MH and practically feasible AMF generators and also devising case-specific treatment techniques.

In principle, MNPs-MH is capable of the non- invasive, remote regulation of cellular/subcellular activities with molecular-level specificity and without limitation in the penetration depth. Based on these characteristic features, more in-depth investigative work is needed to elucidate the biological mechanisms of nanoscale heating extend the target from the single-cell level to the whole body exploited to treat cancer. While MNPs-MH has considerably shown promising future, it is strongly believed that collaborative findings generated from the future works in this MNPs-MH field will enable better design of MNPs for safe and prudent MNPs clinical use.

## Figures and Tables

**Figure 11:**
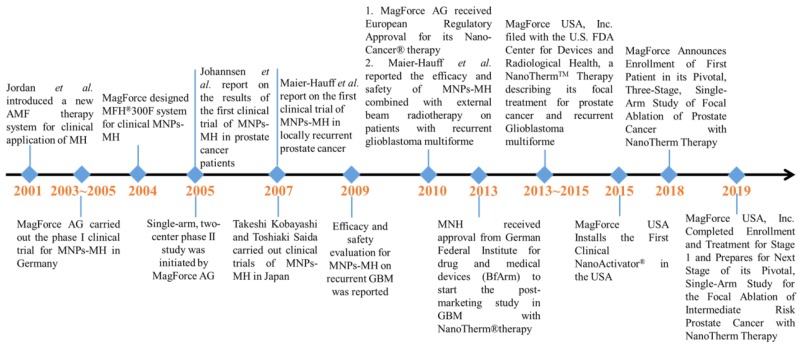
Major events associated with the clinical trials of MNPs-MH.

**Table 1:** Composition and shape effect of MNPs on SAR.

Composition regulation								
Samples	Shape	Size (nm)	*M_s_*(emu/g)	F (kHz)	*H* (kA/m)	SAR/SLP (W/g)	ILP (nHm^2^/kg)	Ref.
Mg_0.13_@γ-Fe_2_O_3_	sphere	7		110	11.14	191	14	25
Fe_0.6_Mn_0.4_O	nanoflowers	102.7	6	366	32	535	1.45	21
Ni_0.8-x_Zn_0.2_Mg_x_Fe_2_O_4_ (x=0)	sphere	36	43.1			-		65
Zn_0.4_Mn0_.6_Fe_2_O_4_	sphere	15	175	500	3.7	432	63	22
Fe_3_O_4_	sphere	22	65	500	15.5	716	5.96	23
MnFe_2_O_4_@CoFe_2_O_4_	core@shell	15	100	500	37.3	3034	4.36	20
CoFe_2_O_4_@Ni_0.5_Zn_0.5_Fe_2_O_4_		9	28.2	265	30	25	0.10	67
Fe@Fe_3_O_4_		13	164	170	26.4	140	1.18	63
FePt@Fe_3_O_4_		15	36	630	18.8	1120	5.03	68
magnetosomes	chains	45.5	81	470	14.4	600	6.16	69
magnetosomes	chains	-	70	108	70.4	1242	2.32	70
magnetosomes	chains	30	-	410	10	960	23.4	30
magnetosomes	chains	40		198	15.2	40	0.87	71
magnetosomes	chains	35	61.4	750	5	171	9.12	72
magnetosomes	chains	45		75	30	375	5.56	74

Shape regulation								
Samples	Shape	Size (nm)	*M_s_*(emu/g)	F (kHz)	*H* (kA/m)	SAR/SLP (W/g)	ILP (nHm^2^/kg)	Ref.
Zn_0.4_Fe_2.6_O_4_	spheres	22	145			-		28
	cubes	18	165	500	37.4	1860	2.66	
Fe_3_O_4_	nanodiscs	125	435	488	47.8	5000	4.48	29
Fe_3_O_4_	nanorings	70	-	400	59.2	3050	2.18	31
CoFe_2_O_4_@Zn_0.4_Fe_2.6_O_4_	core@shell	60	190	500	37.4	10600	15.16	20

## Abbreviations

MH: magnetic hyperthermia
MNPs: magnetic nanoparticles
AMF: alternating magnetic field
MNPs-MH: MNPs-mediated MH
SPIONs: superparamagnetic iron oxide nanoparticles
ROS: reactive oxygen species
MIONs: magnetic iron oxide nanoparticles
SAR: specific heat absorption rate
SLP: specific loss power
ILP: intrinsic loss power
Ms: saturation magnetization
K: magnetocrystalline aniso-tropy
NFs: nanoflowers
FVIOs: ferrimagnetic vortex-domain nanorings
HTS: hypertonic saline
MCNP: magnetic core-shell nanoparticle
ATAP: amphipathic tail-anchoring peptide
PEG: polyethyl-ene glycol
TME: tumor microenvironments
MILH: magnetic intra-lysosomal hyperthermia
RF: radio frequency
BBB: blood-brain barrier
DM-ACMSs: DOX-loaded magnetic alginate/chitosan micro-spheres
RT: radiation therapy
GdIONP: gadolinium-doped iron oxide nanoparticles
MDSCs: myeloid-derived suppressor cells
NPTT: nano-photothermal therapy
Cy7: cyanine7
PES: poly(3,4-ethylenedioxythiophene): poly (4-styrenesulfonate)
PDT: photodynamic therapy

## References

[1] Gilchrist RK, Medal R, Shorey WD, Hanselman RC, Parrott JC, Taylor CB (1957). Selective inductive heating of lymph nodes. Ann Surg.

[2] Blanco-Andujar C, Teran FJ, Ortega D (2018). Chapter 8-Current outlook and perspectives on nanoparticle-mediated magnetic hyperthermia. Mahmoudi M, Laurent S, editors.

[3] Kumar C, Mohammad F (2011). Magnetic nanomaterials for hyperthermia-based therapy and controlled drug delivery. Adv Drug Deliv Rev.

[4] Shubayev VI, Pisanic TR, Jin SH (2009). Magnetic nanoparticles for theragnostics. Adv Drug Deliv Rev.

[5] Yoo D, Lee JH, Shin TH, Cheon J (2011). Theranostic magnetic nanoparticles. Accounts Chem Res.

[6] Ho D, Sun XL, Sun SH (2011). Monodisperse magnetic nanoparticles for theranostic applications. Accounts Chem Res.

[7] Jordan A, Scholz R, Wust P, Schirra H, Thomas S, Schmidt H (1999). Endocytosis of dextran and silan-coated magnetite nanoparticles and the effect of intracellular hyperthermia on human mammary carcinoma cells in vitro. J Magn Magn Mater.

[8] Espinosa A, Kolosnjaj-Tabi J, Abou-Hassan A, Sangnier AP, Curcio A, Silva AKA (2018). Magnetic (hyper)thermia or photothermia? progressive comparison of iron oxide and gold nanoparticles heating in water, in cells, and in vivo. Adv Funct Mater.

[9] Hildebrandt B, Wust P, Ahlers O, Dieing A, Sreenivasa G, Kerner T (2002). The cellular and molecular basis of hyperthermia. Crit Rev Oncol Hemat.

[10] Jordan A, Scholz R, Wust P, Fähling H, Roland F (1999). Magnetic fluid hyperthermia (MFH): cancer treatment with AC magnetic field induced excitation of biocompatible superparamagnetic nanoparticles. J Magn Magn Mater.

[11] Ma M, Wu Y, Zhou J, Sun Y, Zhang Y, Gu N (2004). Size dependence of specific power absorption of Fe_3_O_4_ particles in AC magnetic field. J Magn Magn Mater.

[12] Mehdaoui B, Meffre A, Carrey J, Lachaize S, Lacroix L-M, Gougeon M (2011). Optimal size of nanoparticles for magnetic hyperthermia: a combined theoretical and experimental study. Adv Funct Mater.

[13] Lenaic L, Claudia I, Thangavel K (2011). Water-dispersible sugar-coated iron oxide nanoparticles. An evaluation of their relaxometric and magnetic hyperthermia properties. J Am Chem Soc.

[14] Jeun M, Lee S, Kang JK, Tomitaka A, Kang KW, Kim YI (2012). Physical limits of pure superparamagnetic Fe_3_O_4_ nanoparticles for a local hyperthermia agent in nanomedicine. Appl Phys Lett.

[15] Mohapatra J, Zeng F, Elkins K, Xing M, Ghimire M, Yoon S (2018). Size-dependent magnetic and inductive heating properties of Fe_3_O_4_ nanoparticles: scaling laws across the superparamagnetic size. Phys Chem Chem Phys.

[16] Nemati Z, Alonso J, Martinez LM, Khurshid H, Garaio E, Garcia JA (2016). Enhanced magnetic hyperthermia in iron oxide nano-octopods: size and anisotropy effects. J Phys Chem C.

[17] Lv Y, Yang Y, Fang J, Zhang H, Peng E, Liu X (2015). Size dependent magnetic hyperthermia of octahedral Fe_3_O_4_ nanoparticles. RSC Adv.

[18] Hergt R, Dutz S, Röder M (2008). Effects of size distribution on hysteresis losses of magnetic nanoparticles for hyperthermia. J Phy-Condens Mat.

[19] Rosensweig RE (2002). Heating magnetic fluid with alternating magnetic field. J Magn Magn Mater.

[20] Lee J-H, Jang J-t, Choi J-S, Moon SH, Noh S-h, Kim J-W (2011). Exchange-coupled magnetic nanoparticles for efficient heat induction. Nat Nanotechnol.

[21] Liu XL, Ng CT, Chandrasekharan P, Yang HT, Zhao LY, Peng E (2016). Synthesis of ferromagnetic Fe_0.6_Mn_0.4_O nanoflowers as a new class of magnetic theranostic platform for in vivo T_1_-T_2_ dual-mode magnetic resonance imaging and magnetic hyperthermia therapy. Adv Healthc Mater.

[22] Jang J-T, Nah H, Lee J-H, Moon SH, Kim MG, Cheon J (2009). Critical enhancements of MRI contrast and hyperthermic effects by dopant-controlled magnetic nanoparticles. Angew Chem Int Edit.

[23] Chen R, Christiansen MG, Anikeeva P (2013). Maximizing hysteretic losses in magnetic ferrite nanoparticles via model-driven synthesis and materials optimization. ACS Nano.

[24] Jang J-t, Lee J, Seon J, Ju E, Kim M, Kim YI (2018). Giant magnetic heat induction of magnesium-doped γ-Fe_2_O_3_ superparamagnetic nanoparticles for completely killing tumors. Adv Mater.

[25] Liu X, Peng M, Li G, Miao Y, Luo H, Jing G (2019). Ultrasonication-triggered ubiquitous assembly of magnetic Janus amphiphilic nanoparticles in cancer theranostic applications. Nano Lett.

[26] Du Y, Liu X, Liang Q, Liang X-J, Tian J (2019). Optimization and design of magnetic ferrite nanoparticles with uniform tumor distribution for highly sensitive MRI/MPI performance and improved magnetic hyperthermia therapy. Nano Lett.

[27] Sugumaran PJ, Liu X-L, Herng TS, Peng E, Ding J (2019). GO-functionalized large magnetic iron oxide nanoparticles with enhanced colloidal stability and hyperthermia performance. ACS Appl Mater Inter.

[28] Noh S-h, Na W, Jang J-t, Lee J-H, Lee EJ, Moon SH (2012). Nanoscale magnetism control via surface and exchange anisotropy for optimized ferrimagnetic hysteresis. Nano Lett.

[29] Yang Y, Liu XL, Lv YB, Herng TS, Xu XH, Xia WX (2015). Orientation mediated enhancement on magnetic hyperthermia of Fe_3_O_4_ nanodisc. Adv Funct Mater.

[30] Hergt R, Hiergeist R, Zeisberger M, Schuler D, Heyen U, Hilger I (2005). Magnetic properties of bacterial magnetosomes as potential diagnostic and therapeutic tools. J Magn Magn Mater.

[31] Liu XL, Yang Y, Ng CT, Zhao LY, Zhang Y, Bay BH (2015). Magnetic vortex nanorings: a new class of hyperthermia agent for highly efficient in vivo regression of tumors. Adv Mater.

[32] Xiao W, Liu X, Hong X, Yang Y, Lv Y, Fang J (2015). Magnetic-field-assisted synthesis of magnetite nanoparticles via thermal decomposition and their hyperthermia properties. Crystengcomm.

[33] Liu XL, Fan HM, Yi JB, Yang Y, Choo ESG, Xue JM (2012). Optimization of surface coating on Fe_3_O_4_ nanoparticles for high performance magnetic hyperthermia agents. J Mater Chem.

[34] Huang H, Delikanli S, Zeng H, Ferkey DM, Pralle A (2010). Remote control of ion channels and neurons through magnetic-field heating of nanoparticles. Nat Nanotechnol.

[35] Stanley SA, Gagner JE, Damanpour S, Yoshida M, Dordick JS, Friedman JM (2012). Radio-wave heating of iron oxide nanoparticles can regulate plasma glucose in mice. Science.

[36] Gordon RT, Hines JR, Gordon D (1979). Intracellular hyperthermia. A biophysical approach to cancer treatment via intracellular temperature and biophysical alterations. Med Hypotheses.

[37] Cazares-Cortes E, Cabana S, Boitard C, Nehlig E, Griffete N, Fresnais J (2019). Recent insights in magnetic hyperthermia: From the "hot-spot" effect for local delivery to combined magneto-photo-thermia using magneto-plasmonic hybrids. Adv Drug Deliv Rev.

[38] Sanz B, Calatayud MP, Torres TE, Fanarraga ML, Ibarra MR, Goya GF (2017). Magnetic hyperthermia enhances cell toxicity with respect to exogenous heating. Biomaterials.

[39] Clerc P, Jeanjean P, Hallalli N, Gougeon M, Pipy B, Carrey J (2018). Targeted magnetic intra-lysosomal hyperthermia produces lysosomal reactive oxygen species and causes Caspase-1 dependent cell death. J Control Release.

[40] Domenech M, Marrero-Berrios I, Torres-Lugo M, Rinaldi C (2013). Lysosomal membrane permeabilization by targeted magnetic nanoparticles in alternating magnetic fields. ACS Nano.

[41] Blanco-Andujar C, Walter A, Cotin G, Bordeianu C, Mertz D, Felder-Flesch D (2016). Design of iron oxide-based nanoparticles for MRI and magnetic hyperthermia. Nanomedicine.

[42] Fortin J-P, Wilhelm C, Servais J, Menager C, Bacri J-C, Gazeau F (2007). Size-sorted anionic iron oxide nanomagnets as colloidal mediators for magnetic hyperthermia. J Am Chem Soc.

[43] Hergt R, Dutz S (2007). Magnetic particle hyperthermia-biophysical limitations of a visionary tumour therapy. J Magn Magn Mater.

[44] Jun Y-W, Choi J-S, Cheon J (2007). Heterostructured magnetic nanoparticles: their versatility and high performance capabilities.

[45] Carrey J, Mehdaoui B, Respaud M (2011). Simple models for dynamic hysteresis loop calculations of magnetic single-domain nanoparticles: Application to magnetic hyperthermia optimization.

[46] Kallumadil M, Tada M, Nakagawa T, Abe M, Southern P, Pankhurst QA (2009). Suitability of commercial colloids for magnetic hyperthermia. J Magn Magn Mater.

[47] Kozissnik B, Bohorquez AC, Dobson J, Rinaldi C (2013). Magnetic fluid hyperthermia: Advances, challenges, and opportunity. Int J Hyperther.

[48] Hilger I, Fruhauf K, Andra W, Hiergeist R, Hergt R, Kaiser WA (2002). Heating potential of iron oxides for therapeutic purposes in interventional radiology. Acad Radiol.

[49] Liu XL, Choo ESG, Ahmed AS, Zhao LY, Yang Y, Ramanujan RV (2014). Magnetic nanoparticle-loaded polymer nanospheres as magnetic hyperthermia agents. J Mater Chem B.

[50] Natividad E, Castro M, Mediano A (2009). Adiabatic vs. non-adiabatic determination of specific absorption rate of ferrofluids. J Magn Magn Mater.

[51] Andreu I, Natividad E (2013). Accuracy of available methods for quantifying the heat power generation of nanoparticles for magnetic hyperthermia. Int J Hyperther.

[52] Riedinger A, Guardia P, Curcio A, Garcia MA, Cingolani R, Manna L (2013). Subnanometer local temperature probing and remotely controlled drug release based on azo-functionalized iron oxide nanoparticles. Nano Lett.

[53] Perigo EA, Hemery G, Sandre O, Ortega D, Garaio E, Plazaola F (2015). Fundamentals and advances in magnetic hyperthermia. Appl Phys Rev.

[54] Dutz S, Hergt R, Muerbe J, Mueller R, Zeisberger M, Andrae W (2007). Hysteresis losses of magnetic nanoparticle powders in the single domain size range. J Magn Magn Mater.

[55] Kita E, Oda T, Kayano T, Sato S, Minagawa M, Yanagihara H (2010). Ferromagnetic nanoparticles for magnetic hyperthermia and thermoablation therapy. J Phys D Appl Phys.

[56] Liu XL, Fan HM (2014). Innovative magnetic nanoparticle platform for magnetic resonance imaging and magnetic fluid hyperthermia applications. Curr Opin Chem Eng.

[57] Zhang H, Liu XL, Zhang YF, Gao F, Li GL, He Y (2018). Magnetic nanoparticles based cancer therapy: current status and applications. Sci China Life Sci.

[58] Albarqi HA, Wong LH, Schumann C, Sabei FY, Korzun T, Li X (2019). Biocompatible nanoclusters with high heating efficiency for systemically delivered magnetic hyperthermia. ACS Nano.

[59] Castellanos-Rubio I, Munshi R, Qin Y, Eason DB, Orue I, Insausti M (2018). Multilayered inorganic-organic microdisks as ideal carriers for high magnetothermal actuation: assembling ferrimagnetic nanoparticles devoid of dipolar interactions. Nanoscale.

[60] Castellanos-Rubio I, Rodrigo I, Munshi R, Arriortua O, Garitaonandia JS, Martinez-Amesti A (2019). Outstanding heat loss via nano-octahedra above 20 nm in size: from wustite-rich nanoparticles to magnetite single-crystals. Nanoscale.

[61] Gavilan H, Sanchez EH, Broll MEF, Asin L, Moerner KK, Frandsen C (2017). Formation mechanism of maghemite nanoflowers synthesized by a polyol-mediated process. ACS Omega.

[62] Habib AH, Ondeck CL, Chaudhary P, Bockstaller MR, McHenry ME (2008). Evaluation of iron-cobalt/ferrite core-shell nanoparticles for cancer thermotherapy. J Appl Phys.

[63] Lacroix L-M, Frey Huls N, Ho D, Sun X, Cheng K, Sun S (2011). Stable single-crystalline body centered cubic Fe nanoparticles. Nano Lett.

[64] Lee J-H, Huh Y-M, Jun Y-W, Seo J-W, Jang J-T, Song H-T (2007). Artificially engineered magnetic nanoparticles for ultra-sensitive molecular imaging. Nat Med.

[65] Gabal MA, Bayoumy WA (2010). Effect of composition on structural and magnetic properties of nanocrystalline Ni_0.8-x_Zn_0.2_Mg_x_Fe_2_O_4_ ferrite. Polyhedron.

[66] Yang Y, Liu X, Yang Y, Xiao W, Li Z, Xue D (2013). Synthesis of nonstoichiometric zinc ferrite nanoparticles with extraordinary room temperature magnetism and their diverse applications. J Mater Chem C.

[67] Phadatare MR, Meshram JV, Gurav KV, Kim JH, Pawar SH (2016). Enhancement of specific absorption rate by exchange coupling of the core-shell structure of magnetic nanoparticles for magnetic hyperthermia.

[68] Yang M-D, Ho C-H, Ruta S, Chantrell R, Krycka K, Hovorka O (2018). Magnetic interaction of multifunctional core-shell nanoparticles for highly effective theranostics. Adv Mater.

[69] Plan Sangnier A, Preveral S, Curcio A, K A Silva A, Lefèvre CT, Pignol D (2018). Targeted thermal therapy with genetically engineered magnetite magnetosomes@RGD: Photothermia is far more efficient than magnetic hyperthermia. J Control Release.

[70] Alphandery E, Faure S, Raison L, Duguet E, Howse PA, Bazylinski DA (2011). Heat production by bacterial magnetosomes exposed to an oscillating magnetic field. J Phys Chem C.

[71] Le Fevre R, Durand-Dubief M, Chebbi I, Mandawala C, Lagroix F, Valet J-P (2017). Enhanced antitumor efficacy of biocompatible magnetosomes for the magnetic hyperthermia treatment of glioblastoma. Theranostics.

[72] Timko M, Dzarova A, Kovac J, Skumiel A, Jozefczak A, Hornowski T (2009). Magnetic properties and heating effect in bacterial magnetic nanoparticles. J Magn Magn Mater.

[73] Liang C, Huang S, Zhao W, Liu W, Chen J, Liu H (2015). Polyhedral Fe_3_O_4_ nanoparticles for lithium ion storage. New J Chem.

[74] Muela A, Muñoz D, Martín-Rodríguez R, Orue I, Garaio E, Abad Díaz de Cerio A (2016). Optimal parameters for hyperthermia treatment using biomineralized magnetite nanoparticles: theoretical and experimental approach. J Phys Chem C.

[75] Gandia D, Gandarias L, Rodrigo I, Robles-Garcia J, Das R, Garaio E (2019). Unlocking the potential of magnetotactic bacteria as magnetic hyperthermia agents. Small.

[76] Serantes D, Simeonidis K, Angelakeris M, Chubykalo-Fesenko O, Marciello M, del Puerto Morales M (2014). Multiplying magnetic hyperthermia response by nanoparticle assembling. J Phys Chem C.

[77] Song Q, Zhang ZJ (2004). Shape control and associated magnetic properties of spinel cobalt ferrite nanocrystals. J Am Chem Soc.

[78] Kakwere H, Leal MP, Materia ME, Curcio A, Guardia P, Niculaes D (2015). Functionalization of strongly interacting magnetic nanocubes with (thermo)responsive coating and their application in hyperthermia and heat-triggered drug delivery. ACS Appl Mater Inter.

[79] Martinez-Boubeta C, Simeonidis K, Makridis A, Angelakeris M, Iglesias O, Guardia P (2013). Learning from nature to improve the heat generation of iron-oxide nanoparticles for magnetic hyperthermia applications. Sci Rep-UK.

[80] Liu X-L, Yang Y, Wu J-P, Zhang Y-F, Fan H-M, Ding J (2015). Novel magnetic vortex nanorings/nanodiscs: Synthesis and theranostic applications. Chinese Phys B.

[81] Yang Y, Liu X-L, Yi J-b, Yang Y, Fan H-M, Ding J (2012). Stable vortex magnetite nanorings colloid: Micromagnetic simulation and experimental demonstration. J Appl Phys.

[82] Wang P, Sun J, Lou Z, Fan F, Hu K, Sun Y (2016). Assembly-induced thermogenesis of gold nanoparticles in the presence of alternating magnetic field for controllable drug release of hydrogel. Adv Mater.

[83] Gao F, Zhang T, Liu X, Ghosal A, Wang D, Xie W (2019). Nonmagnetic hypertonic saline-based implant for breast cancer postsurgical recurrence prevention by magnetic field/pH-driven thermochemotherapy. ACS Appl Mater Inter.

[84] Creixell M, Bohorquez AC, Torres-Lugo M, Rinaldi C (2011). EGFR-targeted magnetic nanoparticle heaters kill cancer cells without a perceptible temperature rise. ACS Nano.

[85] Shah BP, Pasquale N, De G, Tan T, Ma J, Lee K-B (2014). Core-shell nanoparticle-based peptide therapeutics and combined hyperthermia for enhanced cancer cell apoptosis. ACS Nano.

[86] Gao L, Zhuang J, Nie L, Zhang J, Zhang Y, Gu N (2007). Intrinsic peroxidase-like activity of ferromagnetic nanoparticles. Nat Nanotechnol.

[87] Ma X, Wang Y, Liu X-L, Ma H, Li G, Li Y (2019). Fe_3_O_4_-Pd Janus nanoparticles with amplified dual-mode hyperthermia and enhanced ROS generation for breast cancer treatment. Nanoscale Horiz.

[88] Dobson J (2008). Remote control of cellular behaviour with magnetic nanoparticles. Nat Nanotechnol.

[89] Bothun GD, Preiss MR (2011). Bilayer heating in magnetite nanoparticle-liposome dispersions via fluorescence anisotropy. J Colloid Interf Sci.

[90] Ruiz-Hernandez E, Baeza A, Vallet-Regi M (2011). Smart drug delivery through DNA/magnetic nanoparticle gates. ACS Nano.

[91] Dias JT, Moros M, del Pino P, Rivera S, Grazu V, de la Fuente JM (2013). DNA as a molecular local thermal probe for the analysis of magnetic hyperthermia. Angew Chem Int Edit.

[92] Ren Y, Zhang H, Chen B, Cheng J, Cai X, Liu R (2012). Multifunctional magnetic Fe_3_O_4_ nanoparticles combined with chemotherapy and hyperthermia to overcome multidrug resistance. Int J Nanomed.

[93] Willerding L, Limmer S, Hossann M, Zengerle A, Wachholz K, ten Hagen TLM (2016). Method of hyperthermia and tumor size influence effectiveness of doxorubicin release from thermosensitive liposomes in experimental tumors. J Control Release.

[94] Alvarez-Berrios MP, Castillo A, Mendez J, Soto O, Rinaldi C, Torres-Lugo M (2013). Hyperthermic potentiation of cisplatin by magnetic nanoparticle heaters is correlated with an increase in cell membrane fluidity. Int J Nanomed.

[95] Tabatabaei SN, Girouard H, Carret A-S, Martel S (2015). Remote control of the permeability of the blood-brain barrier by magnetic heating of nanoparticles: A proof of concept for brain drug delivery. J Control Release.

[96] Hayashi K, Sakamoto W, Yogo T (2016). Smart ferrofluid with quick gel transformation in tumors for MRI-guided local magnetic thermochemotherapy. Adv Funct Mater.

[97] Li J, Hu Y, Hou Y, Shen X, Xu G, Dai L (2015). Phase-change material filled hollow magnetic nanoparticles for cancer therapy and dual modal bioimaging. Nanoscale.

[98] Yao X, Niu X, Ma K, Huang P, Grothe J, Kaskel S (2017). Graphene quantum dots-capped magnetic mesoporous silica nanoparticles as a multifunctional platform for controlled drug delivery, magnetic hyperthermia, and photothermal therapy. Small.

[99] Zhou J, Li J, Ding X, Liu J, Luo Z, Liu Y (2015). Multifunctional Fe_2_O_3_@PPy-PEG nanocomposite for combination cancer therapy with MR imaging. Nanotechnology.

[100] Ullah S, Seidel K, Tuerkkan S, Warwas DP, Dubich T, Rohde M (2019). Macrophage entrapped silica coated superparamagnetic iron oxide particles for controlled drug release in a 3D cancer model. J Control Release.

[101] Jia G, Han Y, An Y, Ding Y, He C, Wang X (2018). NRP-1 targeted and cargo-loaded exosomes facilitate simultaneous imaging and therapy of glioma in vitro and in vivo. Biomaterials.

[102] Xue W, Liu X-L, Ma H, Xie W, Huang S, Wen H (2018). AMF responsive DOX-loaded magnetic microspheres: transmembrane drug release mechanism and multimodality postsurgical treatment of breast cancer. J Mater Chem B.

[103] Rybka JD (2019). Radiosensitizing properties of magnetic hyperthermia mediated by superparamagnetic iron oxide nanoparticles (SPIONs) on human cutaneous melanoma cell lines. Rep Pract Oncol Radiother.

[104] Hosseini V, Mirrahimi M, Shakeri-Zadeh A, Koosha F, Ghalandari B, Maleki S (2018). Multimodal cancer cell therapy using Au@Fe2O3 core-shell nanoparticles in combination with photo-thermo-radiotherapy. Photodiagn Photodyn.

[105] Jiang P-S, Tsai H-Y, Drake P, Wang F-N, Chiang C-S (2017). Gadolinium-doped iron oxide nanoparticles induced magnetic field hyperthermia combined with radiotherapy increases tumour response by vascular disruption and improved oxygenation. Int J Hyperther.

[106] Moros EG, Penagaricano J, NovaK P, Straube WL, Myerson RJ (2010). Present and future technology for simultaneous superficial thermoradiotherapy of breast cancer. Int J Hyperther.

[107] Wang H, Li X, Xi X, Hu B, Zhao L, Liao Y (2011). Effects of magnetic induction hyperthermia and radiotherapy alone or combined on a murine 4T1 metastatic breast cancer model. Int J Hyperther.

[108] Hauser AK, Mitov MI, Daley EF, McGarry RC, Anderson KW, Hilt JZ (2016). Targeted iron oxide nanoparticles for the enhancement of radiation therapy. Biomaterials.

[109] Lin F-C, Hsu C-H, Lin Y-Y (2018). Nano-therapeutic cancer immunotherapy using hyperthermia-induced heat shock proteins: insights from mathematical modeling. Int J Nanomed.

[110] Sato A, Tamura Y, Sato N, Yamashita T, Takada T, Sato M (2010). Melanoma-targeted chemo-thermo-immuno (CTI)-therapy using N-propionyl-4-S-cysteaminylphenol-magnetite nanoparticles elicits CTL response via heat shock protein-peptide complex release. Cancer Sci.

[111] Kobayashi T, Kakimi K, Nakayama E, Jimbow K (2014). Antitumor immunity by magnetic nanoparticle-mediated hyperthermia. Nanomedicine.

[112] Yanase M, Shinkai M, Honda H, Wakabayashi T, Yoshida J, Kobayashi T (1998). Antitumor immunity induction by intracellular hyperthermia using magnetite cationic liposomes. Jpn J Canc Res.

[113] Liu X, Zheng J, Sun W, Zhao X, Li Y, Gong N (2019). Ferrimagnetic vortex nanoring-mediated mild magnetic hyperthermia imparts potent immunological effect for treating cancer metastasis. ACS Nano.

[114] Chao Y, Chen G, Liang C, Xu J, Dong Z, Han X (2019). Iron nanoparticles for low-power local magnetic hyperthermia in combination with immune checkpoint blockade for systemic antitumor therapy. Nano Lett.

[115] Di Corato R, Bealle G, Kolosnjaj-Tabi J, Espinosa A, Clement O, Silva AKA (2015). Combining magnetic hyperthermia and photodynamic therapy for tumor ablation with photoresponsive magnetic liposomes. ACS Nano.

[116] Espinosa A, Bugnet M, Radtke G, Neveu S, Botton GA, Wilhelm C (2015). Can magneto-plasmonic nanohybrids efficiently combine photothermia with magnetic hyperthermia?. Nanoscale.

[117] Das R, Rinaldi-Montes N, Alonso J, Amghouz Z, Garaio E, Garcia JA (2016). Boosted hyperthermia therapy by combined AC magnetic and photothermal exposures in Ag/Fe_3_O_4_ nanoflowers. ACS Appl Mater Inter.

[118] Espinosa A, Di Corato R, Kolosnjaj-Tabi J, Flaud P, Pellegrino T, Wilhelm C (2016). Duality of iron oxide nanoparticles in cancer therapy: amplification of heating efficiency by magnetic hyperthermia and photothermal bimodal treatment. ACS Nano.

[119] Yan H, Shang W, Sun X, Zhao L, Wang J, Xiong Z (2018). "All-in-One" nanoparticles for trimodality imaging-guided intracellular photo-magnetic hyperthermia therapy under intravenous administration. Adv Funct Mater.

[120] Huang W-C, Shen M-Y, Chen H-H, Lin S-C, Chiang W-H, Wu P-H (2015). Monocytic delivery of therapeutic oxygen bubbles for dual-modality treatment of tumor hypoxia. J Control Release.

[121] Curcio A, Silva AKA, Cabana S, Espinosa A, Baptiste B, Menguy N (2019). Iron oxide nanoflowers@CuS hybrids for cancer tri-therapy: Interplay of photothermal therapy, magnetic hyperthermia and photodynamic therapy. Theranostics.

[122] Walther W, Stein U (2009). Heat-responsive gene expression for gene therapy. Adv Drug Deliv Rev.

[123] Ito A, Shinkai M, Honda H, Kobayashi T (2001). Heat-inducible TNF-alpha gene therapy combined with hyperthermia using magnetic nanoparticles as a novel tumor-targeted therapy. Cancer Gene Ther.

[124] Yin PT, Shah S, Pasquale NJ, Garbuzenko OB, Minko T, Lee K-B (2016). Stem cell-based gene therapy activated using magnetic hyperthermia to enhance the treatment of cancer. Biomaterials.

[125] Wang Z, Chang Z, Lu M, Shao D, Yue J, Yang D (2018). Shape-controlled magnetic mesoporous silica nanoparticles for magnetically-mediated suicide gene therapy of hepatocellular carcinoma. Biomaterials.

[126] Wang Z, Zhang F, Shao D, Chang ZM, Wang L, Hu HZ (2019). Janus nanobullets combine photodynamic therapy and magnetic hyperthermia to potentiate synergetic anti-metastatic immunotherapy. Adv Sci.

[127] Thorat ND, Bohara RA, Noor MR, Dhamecha D, Soulimane T, Tofail SAM (2017). Effective cancer theranostics with polymer encapsulated superparamagnetic nanoparticles: combined effects of magnetic hyperthermia and controlled drug release. ACS Biomater Sci Eng.

[128] Tay ZW, Chandrasekharan P, Chiu-Lam A, Hensley DW, Dhavalikar R, Zhou XY (2018). Magnetic particle imaging-guided heating in vivo using gradient fields for arbitrary localization of magnetic hyperthermia therapy. ACS Nano.

[129] Mahmoudi K, Bouras A, Bozec D, Ivkov R, Hadjipanayis C (2018). Magnetic hyperthermia therapy for the treatment of glioblastoma: a review of the therapy's history, efficacy and application in humans. Int J Hyperther.

[130] Wust P, Gneveckow U, Johannsen M, Boehmer D, Henkel T, Kahmann F (2006). Magnetic nanoparticles for interstitial thermotherapy-feasibility, tolerance and achieved temperatures. Int J Hyperther.

[131] Johannsen M, Gneueckow U, Thiesen B, Taymoorian K, Cho CH, Waldofner N (2007). Thermotherapy of prostate cancer using magnetic nanoparticles: Feasibility, imaging, and three-dimensional temperature distribution. Eur Urol.

[132] Johannsen M, Gneveckow U, Taymoorian K, Thiesen B, Waldoefner N, Scholz R (2007). Morbidity and quality of life during thermotherapy using magnetic nanoparticles in locally recurrent prostate cancer: Results of a prospective phase I trial. Int J Hyperther.

[133] Maier-Hauff K, Ulrich F, Nestler D, Niehoff H, Wust P, Thiesen B (2011). Efficacy and safety of intratumoral thermotherapy using magnetic iron-oxide nanoparticles combined with external beam radiotherapy on patients with recurrent glioblastoma multiforme. J Neuro-Oncol.

[134] Maier-Hauff K, Rothe R, Scholz R, Gneveckow U, Wust P, Thiesen B (2007). Intracranial thermotherapy using magnetic nanoparticles combined with external beam radiotherapy: results of a feasibility study on patients with glioblastoma multiforme. J Neuro-Oncol.

[135] Johannsen M, Gneveckow U, Eckelt L, Feussner A, Waldofner N, Scholz R (2005). Clinical hyperthermia of prostate cancer using magnetic nanoparticles: Presentation of a new interstitial technique. Int J Hyperther.

